# AINR: attention-guided implicit neural representations for spatial domain identification in spatial transcriptomics

**DOI:** 10.1093/bioadv/vbag241

**Published:** 2026-08-19

**Authors:** Yusen Zhang, Guodong Xiao, Ponian Li, Jian Liu

**Affiliations:** School of Mathematics and Statistics, Shandong University, Weihai, China

## Abstract

**Motivation:**

Spatial transcriptomics measures gene expression together with spatial locations, but its data are noisy and sparse, and existing graph-based methods are complex and hard to scale.

**Results:**

We present AINR, an end-to-end deep learning framework that models spatial transcriptomics data as a geometrically constrained continuous biological field. AINR combines implicit neural representations with a spatially-aware attention mechanism and a total variation regularization term, using a periodic sine activation function to map spatial coordinates directly to gene expression while preserving spatial smoothness without explicit adjacency matrices. Across six diverse datasets, AINR consistently outperforms existing methods in spatial domain identification and remains robust even under extreme data sparsity.

**Availability and implementation:**

The code for AINR is available at https://github.com/XGD1122/AINR

## 1 Introduction

Human tissues are composed of highly heterogeneous cell types, each performing specific functions to maintain complex physiological systems (Hu, Schroeder *et al.* 2021). Within the tissue microenvironment, cells with similar functions often reside in physical proximity. To link the gene expression profiles of cells with their spatial locations and subsequently dissect the emergent properties and pathological features of tissues at a systems level (Asp *et al.* 2020, Liao *et al.* 2021), spatial transcriptomics (ST) (Marx 2021) technologies have emerged. ST data typically consist of gene expression profiles across various capture spots alongside their corresponding spatial coordinates. Current ST technologies primarily encompass imaging-based methods, such as seqFISH (Eng *et al.* 2019) and MERFISH (Zhang *et al.* 2021), as well as sequencing-based methods like 10x Visium (Ji *et al.* 2020) and Stereo-seq (Chen *et al.* 2022). Notably, sequencing-based techniques often provide more comprehensive genomic coverage. These advancements facilitate the acquisition of gene expression data at spatial resolution, providing powerful tools for investigating spatial patterns of gene expression in complex tissues. However, the rise of ST technologies has introduced novel computational and bioinformatic challenges. First, due to technical bottlenecks in tissue section preparation, RNA capture efficiency, gene expression data are often characterized by high sparsity and significant noise (Liu *et al.* 2021). Consequently, data denoising is typically required before downstream analysis to enhance data quality (Song *et al.* 2023, Tian *et al.* 2024). Second, the spatial heterogeneity of gene expression inherently reflects distinct biological functional regions, raising the challenge of accurately identifying spatial domains from ST data. In addition, recent work such as FineST has sought to address these technical limitations by integrating histology images with spatial gene expression through contrastive learning, achieving nuclei-resolved resolution and an enhanced signal-to-noise ratio (Li *et al.* 2026).

Existing computational strategies, ranging from statistical frameworks (e.g. Giotto, BayesSpace) (Dries *et al.* 2021, Zhao *et al.* 2021) to Graph Neural Network (GNN) (Scarselli *et al.* 2009) models. For example, SpaGCN (Hu, Li *et al.* 2021) constructs an undirected graph by integrating spatial locations and histological images, utilizing a Graph Convolutional Network (GCN) (Kipf and Welling 2017) combined with a deep embedding clustering framework (Xie *et al.* 2016) to identify spatial domains. STAGATE (Dong and Zhang 2022) trains a graph attention autoencoder (Veličković *et al.* 2018) for domain identification. CCST (Li *et al.* 2022) leverage Deep Graph Infomax (Veličković *et al.* 2019) to cluster ST data. SEDR (Xu *et al.* 2024) employs a Variational Graph Autoencoder (VGAE) (Kipf and Welling 2016) with a masked self-supervised learning mechanism to learn spot representations for domain inference. In addition, GraphST (Long *et al.* 2023) utilizes graph contrastive learning to embed gene expression into a latent representation space for spatial clustering. However, their reliance on discrete, pre-defined graphs limits their adaptability to complex tissue geometries and suffers from limited scalability. In addition, although there have been recent attempts to use implicit representations such as GASTON, SUICA, STINR, and GASTON-Mix (Chitra, Arnold *et al.* 2025, Chitra, Dan *et al.* 2025, Luo *et al.* 2025, Zhu *et al.* 2025), they often struggle to balance the capture of high-frequency biological boundaries with the need to suppress random experimental noise, often resulting in either over-smoothing or overfitting to noisy observations.

To overcome these limitations, we propose AINR, an efficient and flexible framework for ST data analysis that re-conceptualizes tissue architecture as a geometrically-constrained continuous biological field. AINR innovatively combines Implicit Neural Representations (INR) (Ben *et al.* 2020) with a Spatially-Aware Attention (SAA) mechanism. (Vaswani *et al.* 2017) and a Total Variation (TV) regularization term, eliminating the need for explicit adjacency matrices while capturing both local and global spatial context. Across six diverse datasets, AINR consistently outperforms existing methods in spatial domain identification and remains robust under extreme data sparsity.

## 2 Material and methods

### 2.1 Datasets and preprocessing

To evaluate the performance of AINR, we employed several publicly available spatial transcriptomics (ST) datasets across diverse biological tissues and technology platforms, including 10x Visium (Ji *et al.* 2020), Stereo-seq (Chen *et al.* 2022), Slide-seqV2 (Stickels *et al.* 2021), STARmap (Wang *et al.* 2018), and osmFISH (Codeluppi *et al.* 2018) (detailed in Table S1). Preprocessing for all datasets was implemented using the Scanpy package (version 1.9.3) (Wolf *et al.* 2018) following a standard pipeline. First, the raw gene expression matrices were filtered to remove low-frequency genes expressed in fewer than three spots. The raw counts were then normalized based on the library size of each spot, followed by a log⁡(1+x) transformation. The resulting gene expression values were further processed using Z-score normalization. For the spatial domain identification task, we utilized Scanpy to identify Highly Variable Genes (HVGs). If the total number of filtered genes exceeded 3000, the top 3000 HVGs were selected to feed into the AINR model; otherwise, all available genes were retained. Considering the sensitivity of sine activation function to the input distribution, the raw spatial coordinates for all spots were linearly scaled and mapped to the range of [-1,1]. This normalization step is critical for accelerating network convergence and enabling the sine activation function to capture fine-grained spatial details within an appropriate frequency range.

### 2.2 Overview of AINR

**Figure 1:**
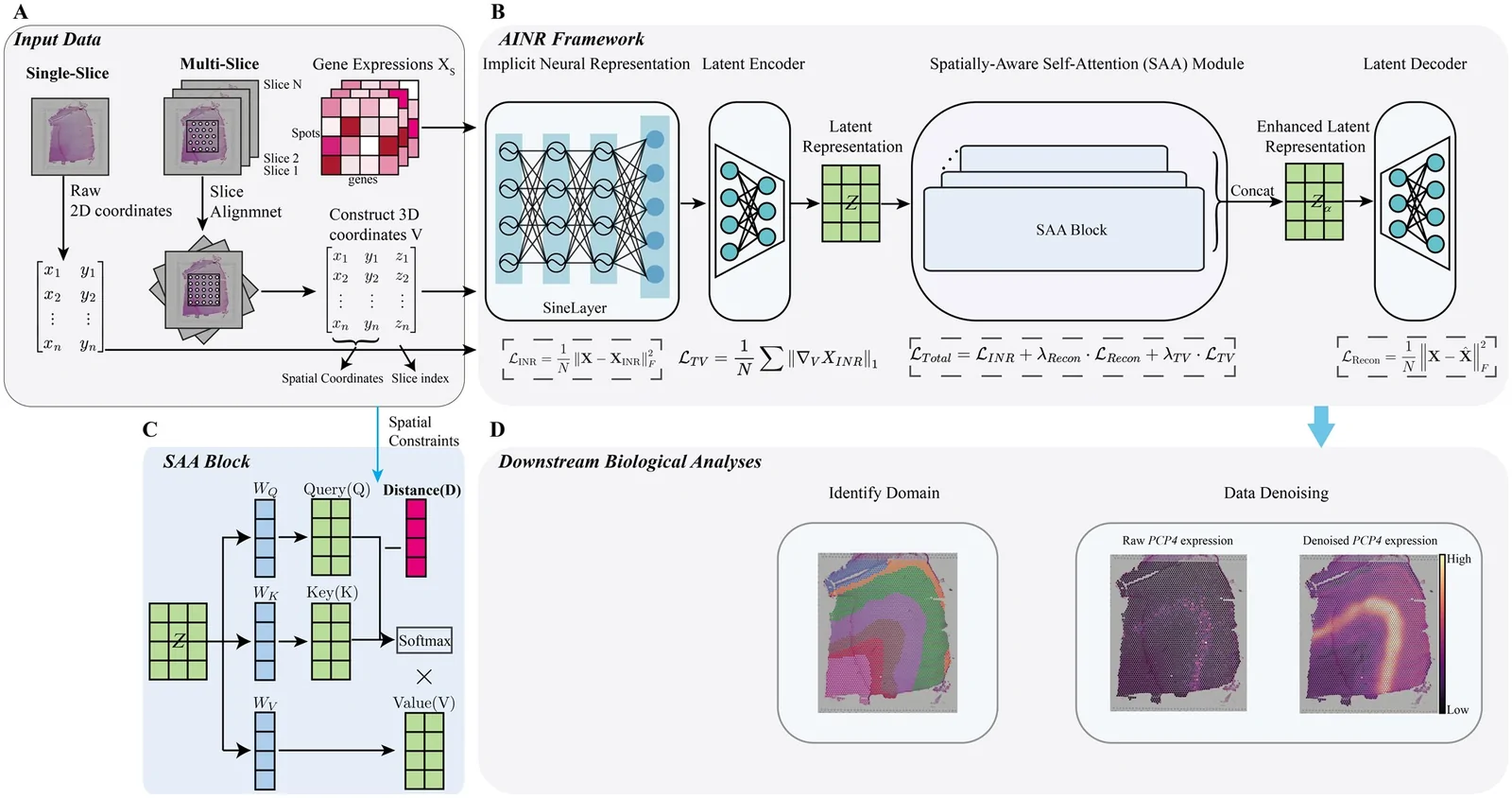
Overview of the AINR framework for spatial transcriptomics (ST) enhancement. (A) Data preprocessing: For multi-slice datasets, individual sections are spatially aligned and normalized within a unified 3D coordinate system; for single-slice data, the original 2D spatial coordinates are used directly, bypassing the slice-index dimension. (B) The spatial coordinates (2D or 3D) are mapped to a latent space via an Implicit Neural Representation (INR) network with periodic sine activation. This representation is further refined by Spatially-Aware Attention blocks to aggregate global contextual information, followed by a Latent Decoder for transcriptomic reconstruction. (C) Detailed architecture of a SAA Block. (D) Downstream analysis: Spatial domain identification and data denoising.

AINR is an end-to-end, continuous, global context-aware deep learning framework designed to effectively resolve spatial transcriptomics (ST) (Marx 2021) data. As illustrated in Fig. 1A, AINR first processes tissue sections through Slice Alignment to construct a unified coordinate matrix V, which incorporates both spatial coordinates and slice indices to maintain anatomical continuity across sections. Subsequently, the framework utilizes this coordinate matrix and the normalized gene expression matrix X as joint inputs (Fig. 1B). Unlike traditional methods that rely on the construction of graphs, AINR leverages an Implicit Neural Representation (INR) (Sitzmann *et al.* 2020) network featuring periodic sine layers to establish a continuous mapping from coordinates to expression space. To maintain Lipschitz-continuous, a Total Variation (TV) regularization term is integrated into the training objective, imposing a rigorous spatial smoothing constraint (Fig. 1B). To further capture global contextual information, the preliminary features are projected into a latent space Z by a Latent Encoder and subsequently processed by a Spatially-Aware Attention (SAA) (Tan *et al.* 2023) module (Fig. 1C). By incorporating a physical distance bias, the SAA module iteratively aggregates global contextual information while prioritizing biologically relevant local neighborhoods. This results in the generation of context-aware, enhanced latent representations $Z_{\propto }$. Subsequently, $Z_{\propto }$ is fed into a Latent Decoder and then mapped back to the original gene expression space to generate the reconstructed matrix$\;\hat{X}$, finally, the outputs of AINR are leveraged for a range of critical downstream biological analyses (Fig. 1D). Specifically, the enhanced latent representations $Z_{\propto }$ are utilized for spatial domain identification, where a Gaussian Mixture Model (GMM) (Scrucca *et al.* 2016) is typically employed. Concurrently, the reconstructed gene expression matrix $\hat{X}$ is used for data denoising.

### 2.3 Spatial coordinate construction and normalization

To fully exploit the biological continuity and intrinsic correlations between adjacent tissue slices, we integrate adjacent slices into a unified 3D coordinate matrix V. In this study, the construction and alignment of 3D spatial coordinates across consecutive tissue slices strictly follow the methodology proposed by Switch3D (Wang *et al.* 2023) (Fig. 1A). For each slice s∈{1,2,.S}, let $X_{S}\in Z^{N_{s}\times G}$ denote the processed gene expression matrix with${\;N}_{s}$ measured spots and G genes. Along with the gene expression, each slice is associated with a coordinate matrix of size $N_{s}\times 2$, which stores the two-dimensional spatial coordinates of the ${\;N}_{s}$ spots. Specifically, we integrate the slice index s as an additional coordinate dimension to obtain a three-dimensional global coordinate matrix $V\in R^{N\times 3}$, where $N=\sum_{s=1}^{S}N_{s}$ is the total number of spots and each row $\left.V\right|(i,:)$ stores the spatial coordinates and the slice index of spot i.

Considering the sensitivity of the sine activation function to the scale of the input distribution, we perform independent min-max normalization on the raw coordinates, linearly mapping them to the [-1,1] interval:


(1) $$\begin{array}{c} {\tilde{v}}_{i}=2\times \frac{v_{i}-v_{\min}}{v_{\max}-v_{\min}+\varepsilon }-1 \end{array}$$

$v_{\min}$ and $v_{\max}$ represent the coordinate boundaries of the dataset, and $\epsilon =10^{-7}$ is a constant to prevent division by zero. This normalization ensures that the sine activation functions can capture fine-grained spatial structures within a reasonable frequency range. For single-slice datasets, the 3D coordinate construction step is bypassed entirely; the original 2D spatial coordinates are used directly as input to the INR network, and the framework operates as a continuous mapping from $R^{2}$ to gene expression space without requiring the slice-index dimension.

### 2.4 INR network

The core of AINR lies in the utilization of a continuous mapping function $f_{\theta }:R^{3}\to R^{G}$ (Fig. 1B), which directly maps the 3D coordinate of each spot to its corresponding gene expression. This process is formulated as:


(2) $$\begin{array}{c} X_{\mathrm{INR}}=f_{\theta }(V) \end{array}$$

where $V\in R^{N\times 3}$ is the global 3D coordinate matrix, $f_{\theta }$ is the INR network mapping function, and $X_{\mathrm{INR}}\in R^{N\times G}$ denotes the Implicit coordinate mapping features. To capture the intricate, high-frequency transcriptomic variations that traditional ReLU-based networks often fail to resolve due to “spectral bias” (Sitzmann *et al.* 2020). The $f_{\theta }$ is defined as a Multi-Layer Perceptron (MLP) utilizing periodic sine activation functions (SIREN). The network architecture is defined as:


(3) $$\begin{array}{c} f_{\theta }(V)≔W_{D}(\;\text{sin}\;(W_{D-1}\ldots \;\text{sin}\;(W_{1}V))) \end{array}$$

where ${\left\{W_{D}\right\}}_{d=1}^{D}$ represents the set of weight matrices and D denotes the depth of the INR, $W_{1}$ (set to 20.0) acts as a frequency multiplier for the first layer to significantly accelerate the extraction of high-frequency features (other set to 1). In addition, to enhance its continuous generalization capability. We introduce a Total Variation (TV) regularization term to enforce spatial smoothness:


(4) $$\begin{array}{c} L_{TV}=\frac{1}{N}\sum_{i=1}^{N}{\left\|\nabla_{v_{i}}f_{\theta }(v_{i})\right\|}_{1} \end{array}$$

where $\nabla_{v_{i}}f_{\theta }(v_{i})$ denotes the spatial gradient of the reconstructed features with respect to the coordinates $v_{i}$. This constraint compels the model to learn a smooth continuous field, effectively mitigating the impact of random noise and dropout events.

We further illustrate this inherent smoothness from the Lipschitz perspective. Let $f_{\theta }$ be the INR mapping function. Since the sine function and its derivatives are bounded, for any 3D coordinates v and $v^{\prime }$, the function satisfies the L-Lipschitz condition:


(5) $$\begin{array}{c} ∥f_{\theta }(v)-f_{\theta }(v^{\prime })∥\leq L∥v-v^{\prime }∥ \end{array}$$

where *L* is the Lipschitz constant. For the sine activation function employed in AINR, |sin’(x)| = |cos(x)| ≤ 1, so each sine layer contributes a factor of at most 1 to the Lipschitz constant. Combined with the TV regularization that directly constrains the spatial gradient norm, the effective Lipschitz constant of the INR is bounded, ensuring stable and smooth interpolation across the spatial domain. This property ensures that the INR naturally preserves smoothness between different spots and across consecutive slices. By leveraging this continuity, AINR implicitly encodes spatial and slice-wise correlations, effectively mitigating the inherent sparsity and noise in ST data without requiring an explicit adjacency graph.

Following the INR mapping, the preliminary spatial features are processed by a Latent Encoder $g_{\omega }$ consisting of MLP layers. This step encodes the $X_{\mathrm{INR}}$ into a latent embedding Z, refining the raw coordinate-based information into a more expressive feature representation for further analysis.


(6) $$Z=g_{\omega }(X_{INR})$$

where $Z\in R^{N\times r}$ (r<G) corresponds to the preliminary latent representations.

### 2.5 Spatially-Aware Attention (SAA) contextual aggregator

Upon obtaining the initial latent representation Z, AINR feeds it into a Spatially-Aware Attention (SAA) (Tan *et al.* 2023) module to facilitate global information integration (Fig. 1C). Traditional self-attention treats spatial spots as a sequence, ignoring their physical proximity. In SAA, we inject a physical distance bias to align the attention mechanism with the underlying tissue geometry. For each head i∈{1,…h}, the attention mechanism is defined as:


(7) $$\begin{array}{c} \mathrm{Attention}(ZW_{i}^{Q},ZW_{i}^{K},ZW_{i}^{V})=\mathrm{softmax}(\frac{Q_{i}K_{i}^{T}}{\sqrt{d_{k}}}-\gamma .\mathrm{Dist})V_{i} \end{array}$$

(8) $$\begin{array}{c} d_{k}=\frac{r}{h} \end{array}$$

where $W_{i}^{Q}$, $W_{i}^{K},W_{i}^{V}\in R^{r\times d_{k}}$ are learnable projection matrices that transform the latent embedding into query (Q), key (K), and value (V) subspaces, respectively. “Dist” is the Euclidean distance matrix between spot coordinates, and γ is a learnable weighting coefficient. By penalizing long-range interactions based on physical distance, the SAA mechanism enables the model to distinguish local correlation and spatially uncorrelated. The scaling factor $\sqrt{d_{k}}$ ensures numerical stability during training. The final latent contextual representation is obtained by concatenating the outputs of all heads and applying a linear projection:


(9) $$\begin{array}{c} Z_{\alpha }=\mathrm{MultiHead}(Z)=\mathrm{Concat}({\mathrm{head}}_{1},\ldots ,{\mathrm{head}}_{h})W^{O} \end{array}$$

where $W^{O}\in R^{r_{d_{k}}\times r}$integrates the diverse attention patterns. In our experiments, h was set to 8. The SAA allows the model to parallelize the capture of global spatial information. Through this fusion, the final latent representation $Z_{\propto }$ is no longer restricted to absolute coordinate mapping; instead, it deeply integrates contextual information from the surrounding microenvironment (Xue *et al.* 2024). This mechanism enables AINR to identify biologically meaningful spatial domains while significantly enhancing the model’s robustness.

Subsequently, the enhanced latent representation $Z_{\propto }$ is fed into a Latent Decoder $h_{\psi }$ (Fig. 1B). The decoder maps the contextual latent representation back to the original gene space to produce the final denoised expression matrix $\hat{X}$:


(10) $$\begin{array}{c} \hat{X}=h_{\psi }(Z_{\alpha }) \end{array}$$

The $h_{\psi }$ are set to sine-activated MLPs. The entire framework is optimized jointly, leveraging both the spatial continuity of the INR and the SAA aggregator to infer spatial domain. The total loss is defined as:


(11) $$\begin{array}{c} L_{\mathrm{total}}=L_{\mathrm{INR}}+\lambda L_{\mathrm{recon}}+\gamma L_{TV} \end{array}$$

(12) $$\begin{array}{c} L_{\mathrm{INR}}=\frac{1}{N}\sum_{i=1}^{N}∥X_{\mathrm{INR},i}-X_{i}{∥}_{2}^{\;2},\;L_{\mathrm{recon}}=\frac{1}{N}\sum_{i=1}^{N}∥{\hat{X}}_{i}-X_{i}{∥}_{2}^{\;2} \end{array}$$

where $L_{\mathrm{INR}}$ compels the INR network to produce spatially coherent gene expression, while $L_{\mathrm{recon}}$ enables the refined representation to preserve global information awareness, and $L_{TV}$ enforces the Lipschitz continuity of the field. The hyperparameter λ and γ regulate the weight of the reconstruction loss and TV loss.

## 3 Results

We compared the performance of AINR against several other algorithms, including CCST, GraphST, DeepST, SEDR, and STAGATE. For all competing methods, we employed the default hyperparameters and clustering algorithms recommended in their respective original publications. The quality of spatial domain identification was evaluated using two widely recognized clustering metrics: the Adjusted Rand Index (ARI) and Normalized Mutual Information (NMI). For the spatial gradient boundary detection benchmark, we additionally employed three metrics: Moran’s I (measuring global spatial autocorrelation of the gradient field), Contrast Ratio (ratio of boundary-region to interior-region gradient magnitude), and AUROC (discrimination between boundary and non-boundary spots).

### 3.1 AINR enhances the accuracy of spatial domain recognition in the human dorsolateral prefrontal cortex

**Figure 2:**
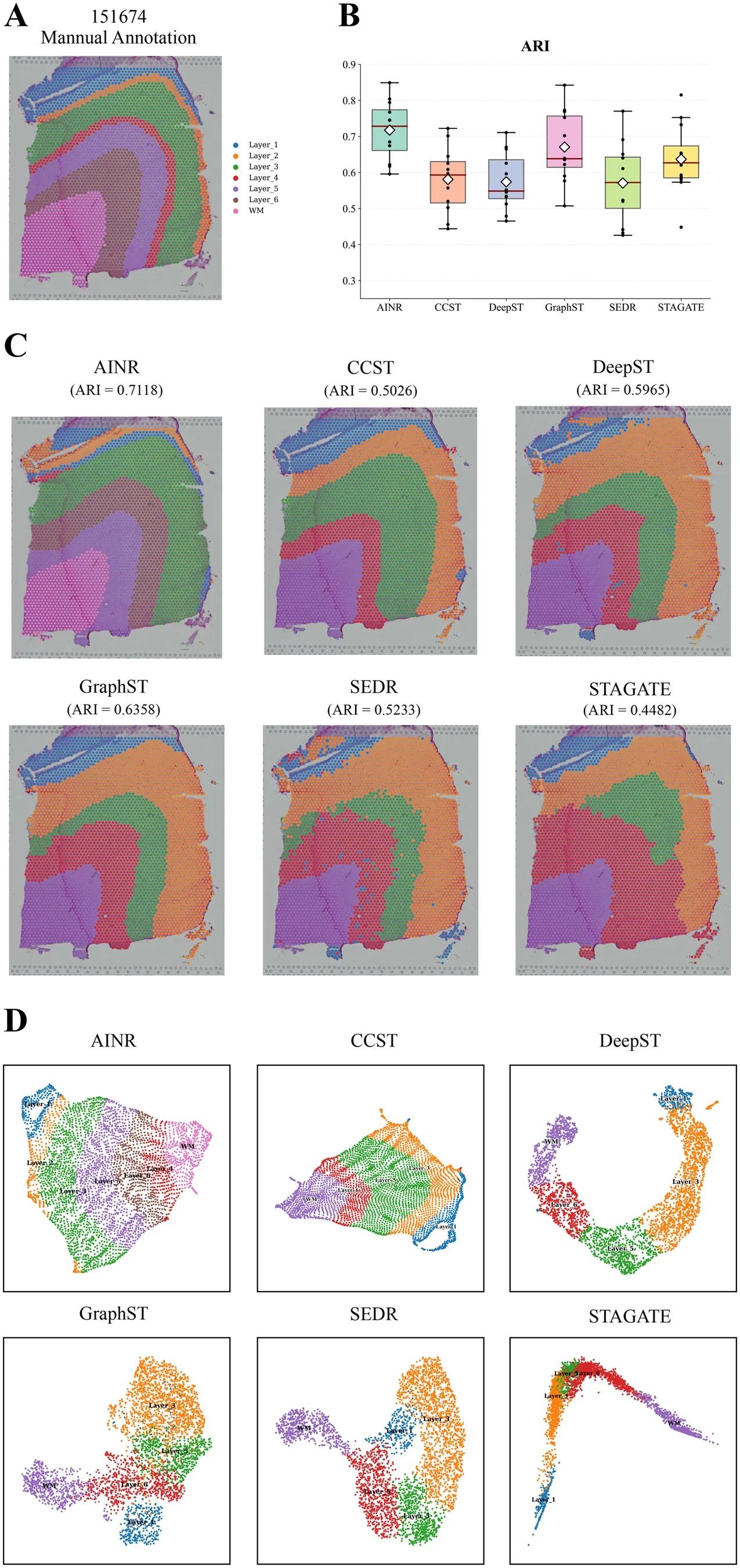
Performance comparison of AINR and baseline methods on the DLPFC dataset. (A) Manual annotations for slice 151 674 of the DLPFC dataset. (B) Boxplots of ARI evaluation results across all 12 slices of the DLPFC dataset for various methods. (C) Example of spatial domain identification results on slice 151 674 by different methods. (D) UMAP visualizations of the reduced embeddings on slice 151 674 for each method.

We evaluated the performance of AINR using manual annotations as the ground truth on the human dorsolateral prefrontal cortex (DLPFC) dataset (Maynard *et al.* 2021), which comprises 12 tissue slices. As illustrated in Fig. 2B, AINR demonstrates superior accuracy and robustness. Specifically, AINR achieved the highest median ARI of 0.72, significantly outperforming the second GraphST (median 0.63) and the third STAGATE (median 0.62). Furthermore, other methods—including SEDR, DeepST, and CCST—struggled to maintain consistent accuracy, with median ARI values falling below 0.6. Their erratic performance across diverse ST datasets highlights a fundamental lack of robustness, whereas AINR demonstrated more consistent performance and sustained accuracy across the tested scenarios. Detailed results for all slices are provided in Figs. S1–S3).

To visually assess the ability of each algorithm to reconstruct cortical laminar structures, we use slice 151 674 as a display (Fig. 2C). Compared to the ground truth (Fig. 2A), the clustering results generated by AINR most closely match manual annotations. Benefiting from the integration of Implicit Neural Representations and Multi-head self-attention mechanisms, AINR correctly identifies the white matter (WM) and other cortical layers (L1–L6) with clear and smooth boundaries. In contrast, SEDR and STAGATE performed poorly on this slice (ARI of 0.52 and 0.45). As shown in Fig. 2C, these methods could only separate WM and L1, while the identification of the remaining layers suffered from severe cluster mixing. CCST lacked the spatial precision required to distinguish L3 from L6, resulting in blurred intermediate layers and a failure to identify L2. Although GraphST and DeepST achieved some degree of separation, they struggled to accurately depict the physical thickness of the layers. Specifically, DeepST erroneously expanded the width of L3 and mixed L2 with L3, while GraphST exhibited disproportionate layer thicknesses, failing to restore the refined anatomical proportions of the cortex. In summary, AINR excels in maintaining domain consistency and restoring anatomical details, overcoming the limitations of existing methods in boundary delineation and thickness prediction.

AINR integrates Implicit Neural Representation (INR) with Attention mechanism, generating a more expressive representation. This representation is visualized using UMAP (Healy and McInnes 2024) (Fig. 2D). AINR generated well-separated clusters that capture the relative locations of different spatial domains. In contrast, GraphST and SEDR exhibited significant inter-layer overlap, leading to blurred spatial boundaries. Other methods (CCST, DeepST, and STAGATE) failed to effectively resolve adjacent layers, resulting in disordered distributions. These results confirm that AINR extracts superior, biologically meaningful representations that facilitate spatial domain identification.

### 3.2 Spatial gene denoising

Raw spatial transcriptomics (ST) data often suffer from low capture efficiency, leading to high noise levels and severe “dropout” events that obscure spatial expression patterns. AINR leverages the properties of Implicit Neural Representations to model gene expression as a continuous biological field, enabling the recovery of high-fidelity expression maps from sparse observations.

**Figure 3:**
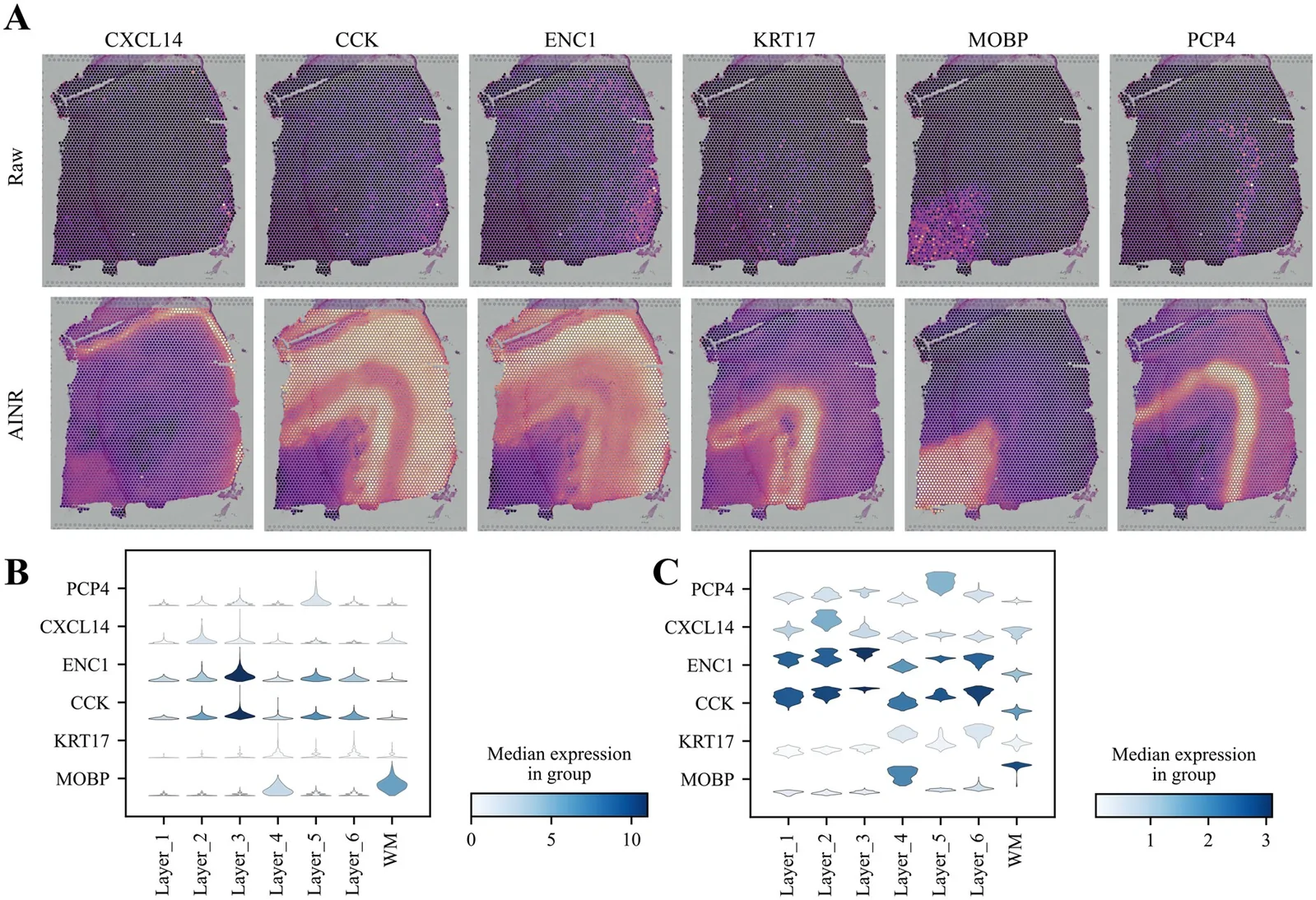
Gene expression denoising by AINR on the DLPFC Dataset. (A) Visualizations between raw (top row) and AINR denoised (bottom row) spatial expressions for six layer-specific marker genes (CXCL14, CCK, ENC1, KRT17, MOBP, and PCP4) in the DLPFC section 151 674. (B) Violin plots of the raw expression of layer-marker genes (C). Violin plots of AINR-denoised layer-marker gene expressions, with corresponding cortical layers highlighted in red.

Using slice 151 674 as an example, we selected six representative layer-specific marker genes: *CXCL14* (L2), *CCK* (L2/3), *ENC1* (L2/3), *KRT17* (L6), *MOBP* (WM), and *PCP4* (L5). As shown in Fig. 3A, the signal in the raw data is extremely sparse and fragmented, making it difficult to discern clear anatomical boundaries. In contrast, the expression maps reconstructed by AINR exhibit remarkable continuity and anatomical accuracy. Crucially, the reconstructed patterns are not mere interpolations but are anchored by the observed data and constrained by the Lipschitz-continuous field. For instance, the expression of *PCP4* is significantly enriched in Layer 5, consistent with previously reported findings (Zeng *et al.* 2012).

Furthermore, we utilized violin plots to visualize the differences in marker gene expression across layers between the raw data (Fig. 3B) and the AINR-reconstructed data (Fig. 3C). In the raw expression, marker genes are confounded by background noise and low median intensity within target layers. Conversely, the violin distributions in the AINR-processed data demonstrate enhanced layer specificity for each marker gene. This highlights the potential of AINR as a robust preprocessing tool for spatial transcriptomics to enhance the reliability of downstream biological discovery. It should be noted that AINR is not a generative model that fabricates patterns from a learned distribution; rather, it is a constrained optimization framework where the reconstruction is anchored by observed values through the reconstruction losses and bounded by Lipschitz continuity, ensuring that the recovered spatial patterns represent the continuous manifold rather than spurious “hallucinations.”

### 3.3 Identification of laminar structures in the mouse olfactory bulb

**Figure 4:**
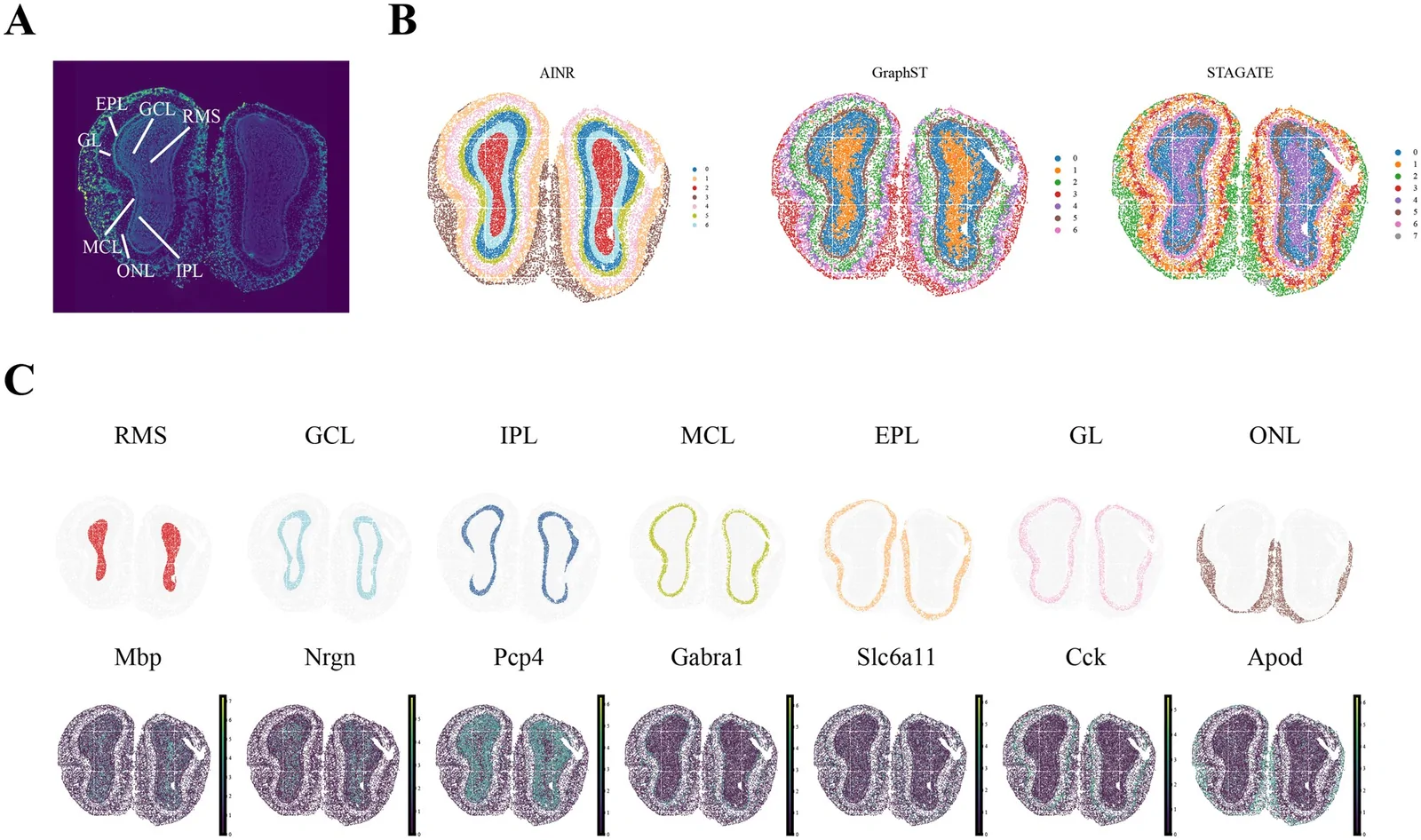
Spatial domains identification on the Stereo-seq Mouse Olfactory Bulb (MOB) dataset. (A) The laminar structure of the Stereo-seq MOB annotated in DAPI-stained images. (B) Comparison of spatial domain identification results between AINR, GraphST, and STAGATE. (C) Visualization of individual spatial domains identified by AINR (top) and their corresponding marker gene expression (bottom).

Subsequently, we evaluated the performance of AINR alongside GraphST and STAGATE on a mouse olfactory bulb (MOB) dataset acquired via Stereo-seq. Based on the DAPI staining image (Fig. 4) (Xu *et al.* 2024), we annotated seven distinct layers of the mouse olfactory bulb. From the internal to the external layers, these are: the rostral migratory stream (RMS), granule cell layer (GCL), internal plexiform layer (IPL), mitral cell layer (MCL), external plexiform layer (EPL), glomerular layer (GL), and olfactory nerve layer (ONL) (Fig. 4A).

Comparative benchmarking (Fig. 4B) highlights AINR’s superior performance in capturing the MOB stratification. While all methods (AINR, GraphST, and STAGATE) successfully outlined the global morphology, their performance in fine-grained structural partitioning differed substantially. The structures produced by STAGATE and GraphST lacked clarity, with severe erroneous mixing between different spatial domains and blurred boundaries (see Fig. S4). Specifically, STAGATE failed to delineate continuous layers, merging the GCL with the IPL and showing extensive overlap between the EPL and GL. Although GraphST slightly improved upon STAGATE, it erroneously clustered cells from the MCL, EPL, and GL into a single domain. In contrast, AINR successfully overcame the interference of noise, resolving all seven layers—including the narrow internal RMS and IPL—with high continuity and sharp boundaries that are highly consistent with the anatomical stratification.

We compared the distribution of marker genes with the corresponding domains identified by AINR to further validate its clustering results (Fig. 4C). Most clustering regions aligned well with the corresponding marker genes. For example, *Mbp* precisely corresponds to the RMS region, *Gabra1* identifies the MCL. Notably, the observed expression overlap of markers like *Mbp* and *Pcp4* in adjacent regions aligns with biological reality, as adjacent internal structures of an organ often share cell types or exhibit transitional states. In summary, AINR achieves high-fidelity spatial domain identification while maintaining biological relevance, providing a scalable tool for resolving intricate tissue structures at subcellular or high-resolution scales.

### 3.4 AINR resolves complex structures of the mouse hippocampus

**Figure 5:**
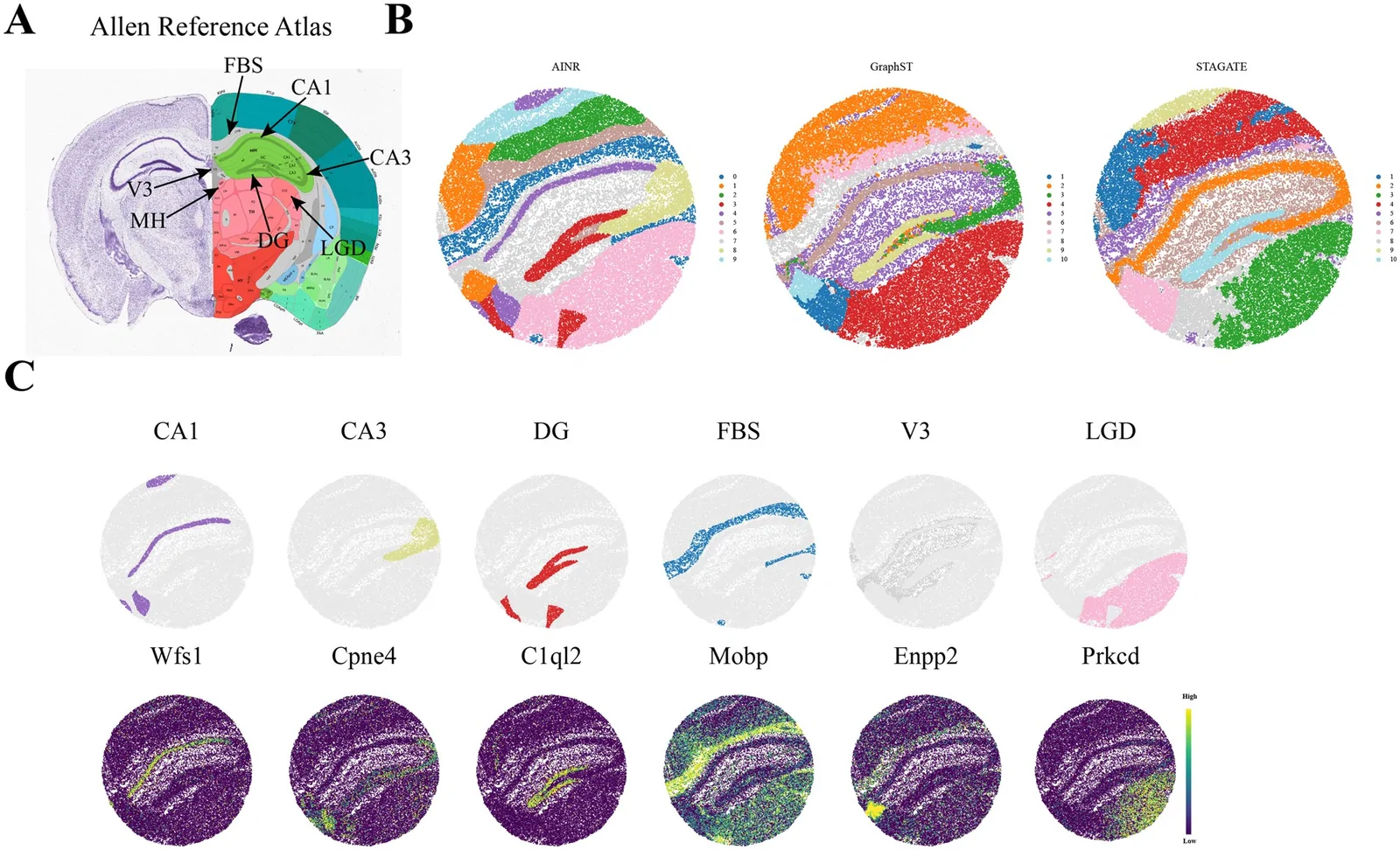
AINR clustering improves the identification of tissue structures in the mouse hippocampus tissue. (A) Allen Mouse Brain Atlas with the hippocampus region annotated. (B) Spatial domains identification results between AINR, GraphST, and STAGATE in mouse hippocampus tissue acquired with Slide-seq V2. (C) Visualization of spatial domains identified by AINR in mouse hippocampus tissue and the corresponding expression patterns of marker genes.

To further evaluate the performance of AINR in resolving brain tissues with highly complex architectures, we compared with GraphST and STAGATE using the mouse hippocampus dataset. In this experiment, we utilized the Allen Brain Atlas as the objective ground-truth (Fig. 5A). As shown in Fig. 5B, Although the global anatomical architecture of the hippocampus was identifiable by all methods, AINR exhibited a distinct advantage in resolving its intricate sub-regions. By leveraging its continuous representation, AINR yielded sharper, more biologically-consistent boundaries compared to the fragmented patterns observed in GraphST and STAGATE. Specifically, GraphST suffered from stray points, leading to blurred transitions between structures. Although STAGATE partially mitigated this noise showed structural deviations, such as irregular fusions within internal hippocampal regions and a failure to isolate peripheral tissues. In contrast, AINR demonstrated superior spatial continuity and structural fidelity, precisely delineated the fiber bundle system (FBS) and the classical hippocampus subregions, including the CA1, CA3, and the dentate gyrus (DG). AINR was the only method capable of clearly depicting the shape and position of the third ventricle (V3). Conversely, the corresponding regions in the GraphST results were dominated by noise, and STAGATE failed to reconstruct the independent morphology of these two structures. Additionally, AINR successfully identified the lateral geniculate complex (LGD).

Validation using marker genes (Fig. 5C) confirmed the biological accuracy of AINR’s spatial domains. The results show a high degree of spatial consistency between the AINR-defined domains and the enrichment areas of these marker genes. Specifically, the shapes of the CA1, CA3, and DG subregions perfectly match the high-expression regions of *Wfs1*, *Cpne4*, and *C1ql2*, respectively. The expression of *Mobp* precisely maps to the FBS region, while *Enpp2* expression validates the positions of V3. Furthermore, the distribution of *Prkcd* aligns closely with the LGD domain identified by AINR. These findings underscore AINR’s capacity to robustly mitigate spatial noise and learn highly expressive latent features, thereby resolving complex tissue domains.

### 3.5 Deciphering spatial heterogeneity in breast cancer with AINR

**Figure 6:**
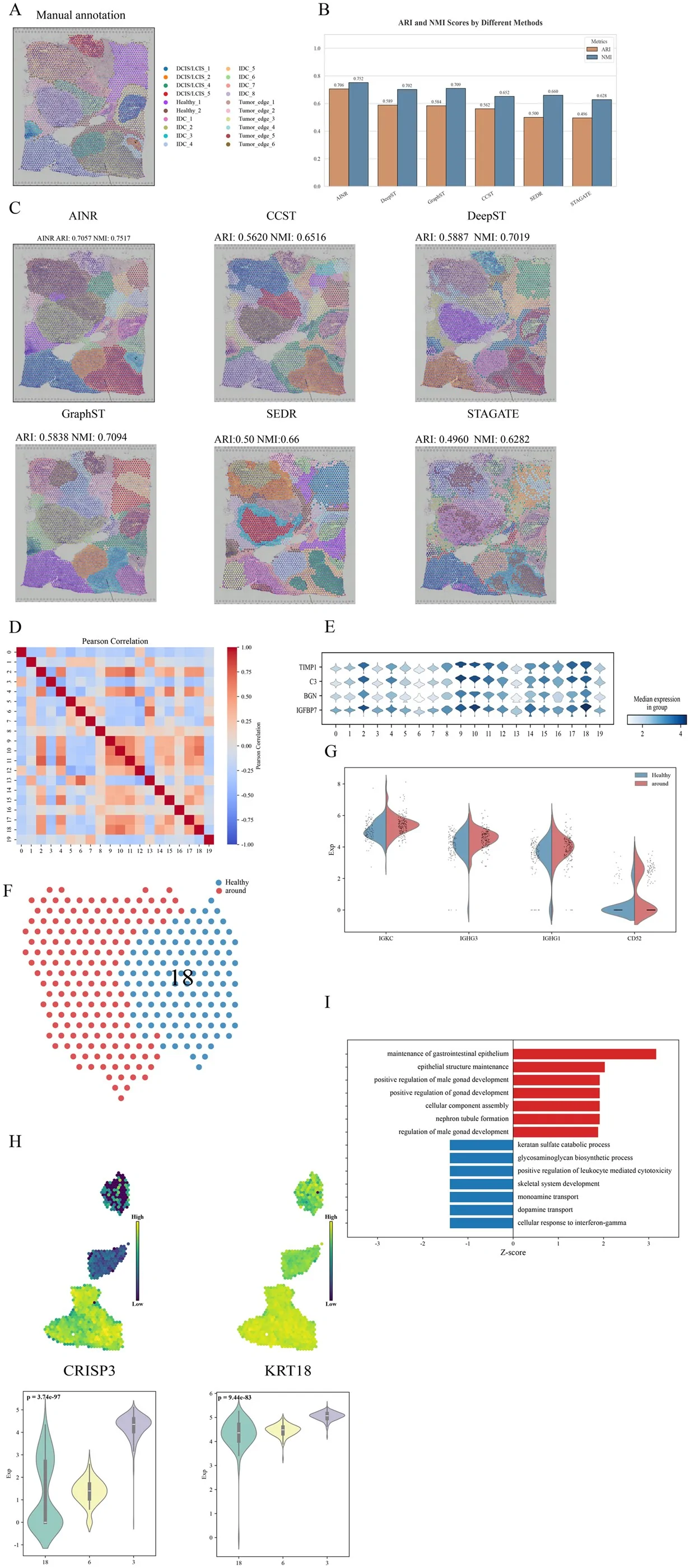
Clustering Performance and Downstream Analyses on the BRCA Dataset. (A) Manual annotations for the BRCA dataset. (B) Boxplots comparing ARI and NMI scores across different methods on the BRCA dataset. (C) Visual comparison of spatial clustering results across different methods. (D) Pearson correlation heatmap of gene expression across different spatial domains, where Domains 9 and 10 exhibit significant heterogeneity compared to other domains. (E)Violin plots of marker gene expression in Domains 9 and 10. (F) Region of the domain 18 (healthy) and around. (G) Differential expression analysis among domains 18 and around. (H) Differential gene expression analysis (top) and violin plots (bottom) comparing Healthy (Domain 18), DCIS/LCIS (Domain 6), and IDC (Domain 3). (I) Gene ontology enrichment analysis of the DEGs between domains 3 and 6. Red denotes pathway with upregulated genes in domain 6; blue is the opposite.

Then we benchmarked it on a human breast cancer (BRCA) dataset against diverse baseline methods. AINR’s identified domains align closely with manual histological annotations (Xu *et al.* 2024) (Fig. 6A). In contrast, STAGATE suffers from local noise, producing fragmented spots within the same pathological region. SEDR and DeepST exhibited blurred boundaries and erroneous merging when distinguishing the tumor core from the adjacent regions, while CCST and GraphST produced large-scale misclassifications (Fig. 6C). Quantitatively, AINR achieved the highest ARI and NMI scores (Fig. 6B), underscoring its superior in identifying spatial domains within highly heterogeneous tumor microenvironments.

To assess spatial heterogeneity within the breast cancer microenvironment, we investigated Domains 9 and 10, which exhibited distinct transcriptomic divergence from other tissue regions (Fig. 6D). Differential expression analysis (Fig. 6E) revealed significant enrichment of several malignancy-associated markers within these domains, notably *IGFBP7* and *TIMP1*. High levels of tumor-specific *IGFBP7* have been shown to interact with membrane-bound InsR, significantly increasing the risk of distant metastasis and general breast cancer progression events (Godina *et al.* 2025). Concurrently, extracellular vesicle-associated *TIMP1* is markedly upregulated in breast cancer tissues compared to normal tissues, where its elevated expression is tightly linked to tumor cell proliferation, extracellular matrix remodeling, and pathological transitions (Ayan *et al.* 2025).

Furthermore, we utilized our framework to resolve the tumor-associated spatial transitions at the invasive margins by comparing the healthy tissue of Domain 18 with the tumor-impacted peripheral regions of Domains 14 and 10 (Fig. 6F). This spatial partitioning revealed prominent differences in oncogenic and microenvironmental markers. Differential expression analysis indicated a strong enrichment of humoral and immune-related genes in these peripheral domains, including *IGKC*, *IGHG1*, and *CD52* (Fig. 6G). *IGKC* is recognized as a robust prognostic indicator in several solid malignancies, including breast cancer (Pandey *et al.* 2014). *IGHG1* is typically overexpressed in highly aggressive phenotypes and correlates with dismal survival; experimental evidence demonstrates that its knockdown significantly impairs cell viability, invasion, and epithelial-mesenchymal transition (EMT) via the inhibition of MEK/ERK and AKT pathways (Jin *et al.* 2023). Additionally, CD52 is significantly upregulated in these boundary zones. As an essential factor in maintaining an immune-dominant tumor microenvironment, CD52 expression heavily correlates with the recruitment of CD8+ T cells and M1 macrophages, acting as a potential prognostic biomarker and therapeutic target. The localized expression of these immune-modulatory and malignancy-associated markers at the tissue boundary (Fig. 6G) provides key spatial evidence of localized invasion and immune-stromal remodeling.

Finally, we characterized the evolutionary transition from healthy tissue (Domain 18) to ductal carcinoma in situ (DCIS, Domain 6) and invasive ductal carcinoma (IDC, Domain 3) (Fig. 6H). In the transition from healthy to invasive states, markers associated with hypoxic survival and metastasis were progressively enriched (Fig. 6H). Notably, *CRISP3* and *KRT18* exhibited significant spatial enrichment. *CRISP3* is frequently upregulated during tumor progression and has been shown to actively promote malignant breast cancer phenotypes under hypoxic conditions by triggering the IL-17/AKT signaling pathway (Ren *et al.* 2025). Concurrently, cytokeratin *KRT18* is a prominent metastatic biomarker classically detected in circulating tumor cells (CTCs), further highlighting the acquisition of metastatic potential during the invasive transition (Khoshbakht *et al.* 2021). To explore the biological pathways separating the non-invasive and invasive stages, Gene Ontology (GO) enrichment analysis was performed to functionally differentiate Domain 6 (DCIS, enriched in red-labeled pathways) and Domain 3 (IDC, enriched in blue-labeled pathways) (Fig. 6I). These transitional pathways and marker profiles demonstrate the capacity of our framework to resolve fine-grained spatial trajectories of malignant evolution and microenvironmental adaptation.

### 3.6 AINR effectively captures the structure in both the mouse visual cortex STARmap dataset and the mouse somatosensory cortex osmFISH dataset

**Figure 7:**
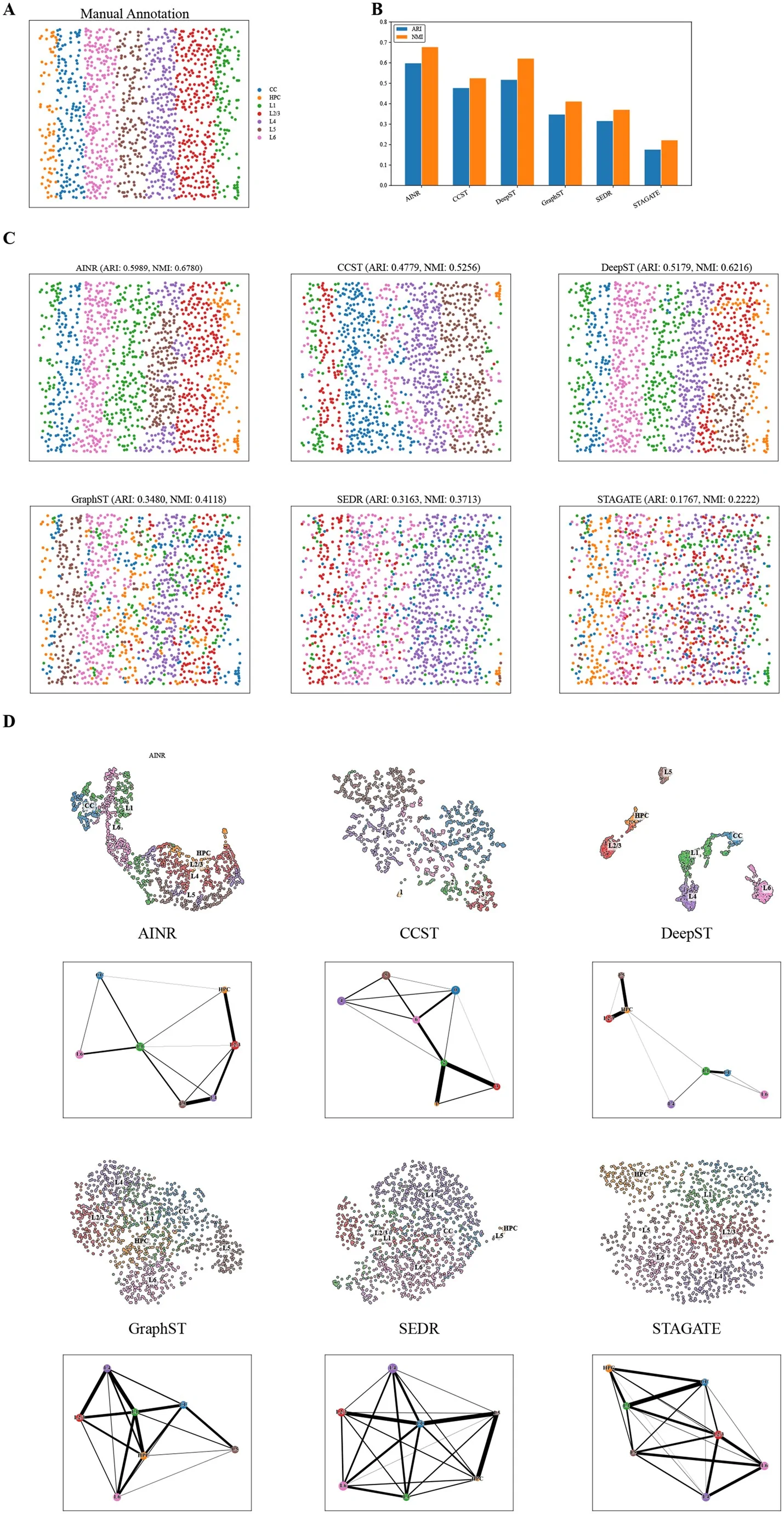
Spatial domains identification on the STARmap MVC dataset. (A)Manual annotation of the STARmap MVC. (B) ARI and NMI bar charts for six methods. (C) The spatial domains identified by AINR, CCST, DeepST, GraphST, SEDR and STAGATE. (D) UMAP visualization and PAGA graph generated by the embedding of these methods.

Finally, we tested the performance of AINR on the mouse visual cortex (MVC) STARmap dataset (Fig. 7) with low gene expression capture rates. Despite technical constraints, AINR’s spatial domains precisely aligned with the ground truth (L1–L6, cc, and HPC) (Fig. 7A), yielding the most continuous boundaries among all methods (Fig. 7C). AINR also achieved superior ARI and NMI scores (Fig. 7B). UMAP visualization and PAGA trajectory inference further demonstrated that AINR’s latent representations maintain high cluster separation and accurately reconstruct the linear trajectory from superficial to deep layers (Fig. 7D). In contrast, GraphST and STAGATE produced fragmented clusters and failed to reflect the structure.

AINR was also employed to the mouse somatosensory cortex (MSC) osmFISH dataset, which targets only 33 genes (Fig. S5). Even with such extreme sparsity, AINR clearly isolated the Pia Layer 1 and other layers (L5, L6) (Fig. S5C). While competing methods suffered from dispersed clustering. AINR significantly outperformed them in all quantitative metrics (Fig. S5B). These results underscore AINR’s unique ability to leverage global spatial context to overcome limited genetic information, achieving topologically meaningful clustering where traditional methods falter.

### 3.7 Ablation study of AINR components

To evaluate the contribution of AINR’s core components, we conducted comprehensive ablation experiments using the 12-slice human DLPFC dataset. We compared the full framework (AINR-FULL) against six architectural variants:

- AINR-w/o SAA: It removes the SAA module to test the impact of global context aggregation.
- AINR-ReLU: It replaces the periodic sine activation function with a standard ReLU function to evaluate the necessity of high-frequency spatial mapping.
- AINR-w/o $L_{\mathrm{Recon}}$: It removes the reconstruction loss to determine the importance of Latent Decoder.
- AINR-w/o $L_{TV}$: It removes the TV loss.
- Single-slice: It processes data slice-by-slice without 3D alignment to evaluate the value of constructing the 3D matrix.
- INR + PCA: It replaces the SAA module and Latent Decoder with PCA (top 50 principal components) directly applied to the INR output features.

**Figure 8:**
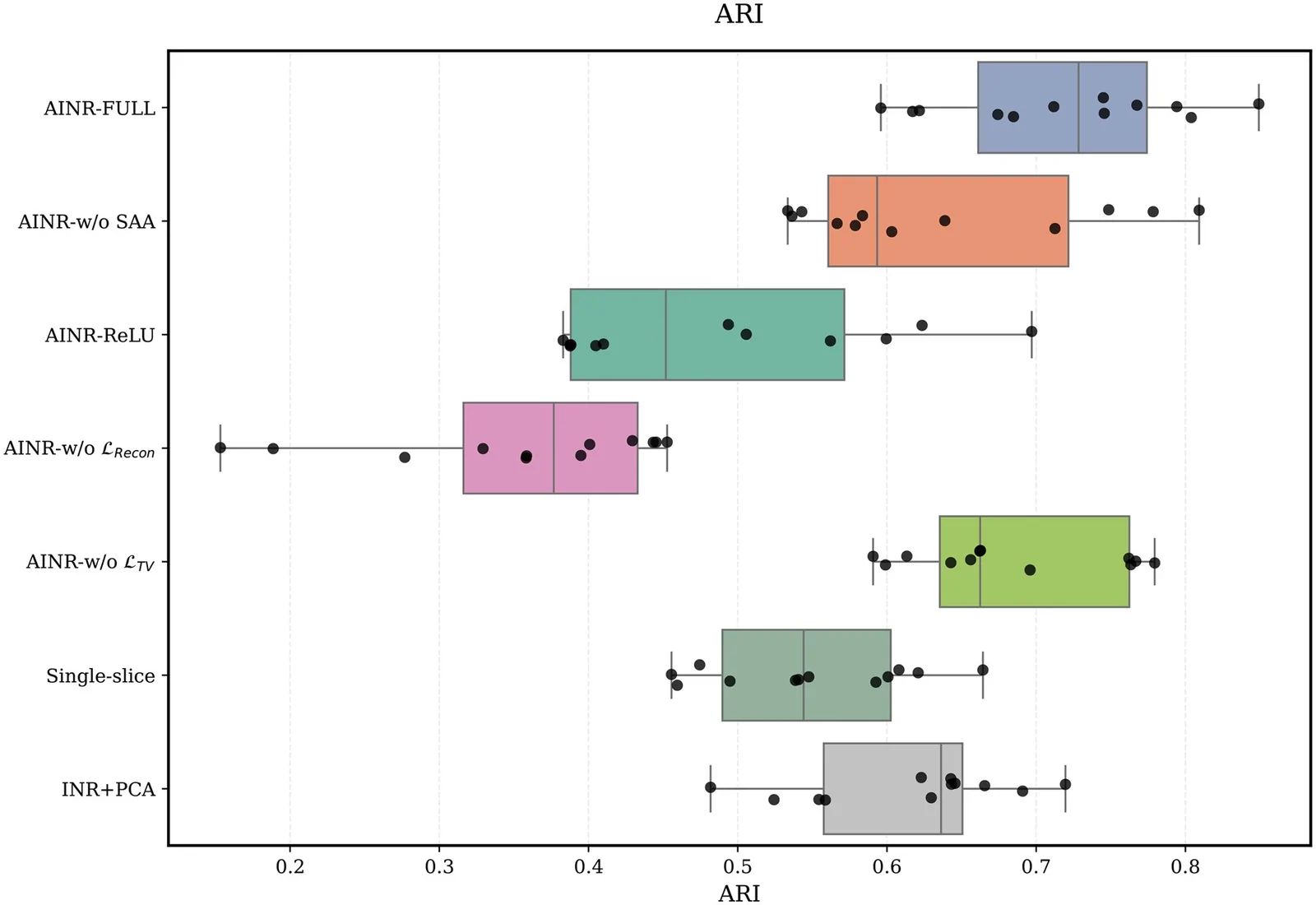
The ARI boxplots of AINR and its variants on the DLPFC dataset.

As illustrated in Fig. 8, AINR-FULL achieved the highest median ARI score. The removal of the reconstruction loss (AINR-w/o $L_{\mathrm{Recon}}$) resulted in the most dramatic performance degradation, with the median ARI dropping below 0.4. Meanwhile, the omission of the TV loss also led to a clear decline in median ARI compared to AINR-FULL, demonstrating that spatial smoothing regularization is essential for robust and accurate domain partitioning. highlighting that Latent Decoder is the primary driver of domain identification. Furthermore, the AINR-ReLU variant exhibited a severe decline in accuracy, confirming that traditional ReLU functions are inadequate for capturing the high-frequency spatial gradients inherent in ST data. While AINR-w/o SAA and Single-slice maintained moderate performance, they were consistently inferior to the full model, validating the essential role of SAA and 3D Slice integration in spatial domain identification. The INR + PCA variant achieved a mean ARI of approximately 0.615 across all 12 DLPFC slices, outperforming the AINR-w/o $L_{\mathrm{Recon}}$ variant but falling below the full model, confirming that while the INR backbone provides effective spatial smoothing, the SAA and Decoder components contribute substantially to domain identification accuracy.

### 3.8 Impact of dropout rates on spatial domain identification

**Figure 9:**
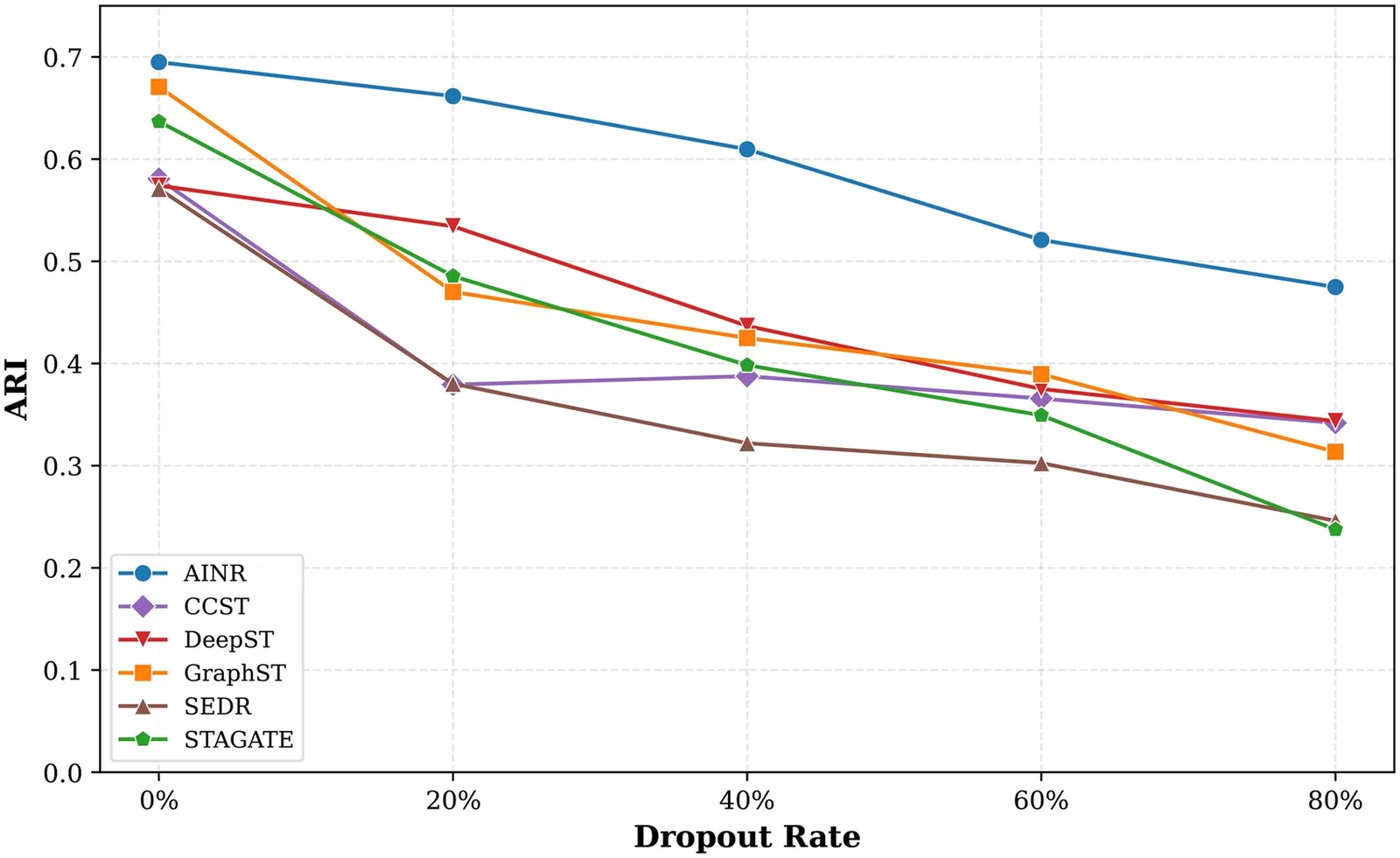
The average clustering performance of AINR and five comparison methods varies with the dropout rate across four slices (151 507–151 510) of the DLPFC dataset.

Spatial transcriptomics data are inherently susceptible to severe technical noise and gene “dropout” events caused by limited capture efficiency. To evaluate the robustness of AINR under varying degrees of data sparsity, we simulated extreme dropout conditions by randomly masking non-zero gene expression values at rates ranging from 20% to 80%. We evaluated AINR with the TV loss removed against five baseline methods using the average ARI across four DLPFC slices (151 507–151 510). As shown in Fig. 9, AINR outperformed all baseline methods across the entire spectrum of dropout rates. As the dropout rate increased, the performance of baseline methods deteriorated rapidly. In contrast, AINR demonstrates exceptional robustness to data loss. Even at the 80% dropout threshold, AINR maintained average ARI of approximately 0.48, significantly surpassing the second method. This exceptional stability indicates that AINR’s architecture—specifically the continuous spatial smoothing provided by the INR combined with the global contextual from the SAA—can effectively recover true biological signals from highly fragmented and sparse gene expression. This analysis serves as a stress test of algorithmic resilience to data sparsity rather than a statistical model of zero-inflation; by demonstrating recovery from extreme dropout, we show that the continuous representation paradigm can maintain biological fidelity even under conditions far more severe than typically encountered in real ST data.

### 3.9 Validation of continuous field properties and biological boundary detection

**Figure 10:**
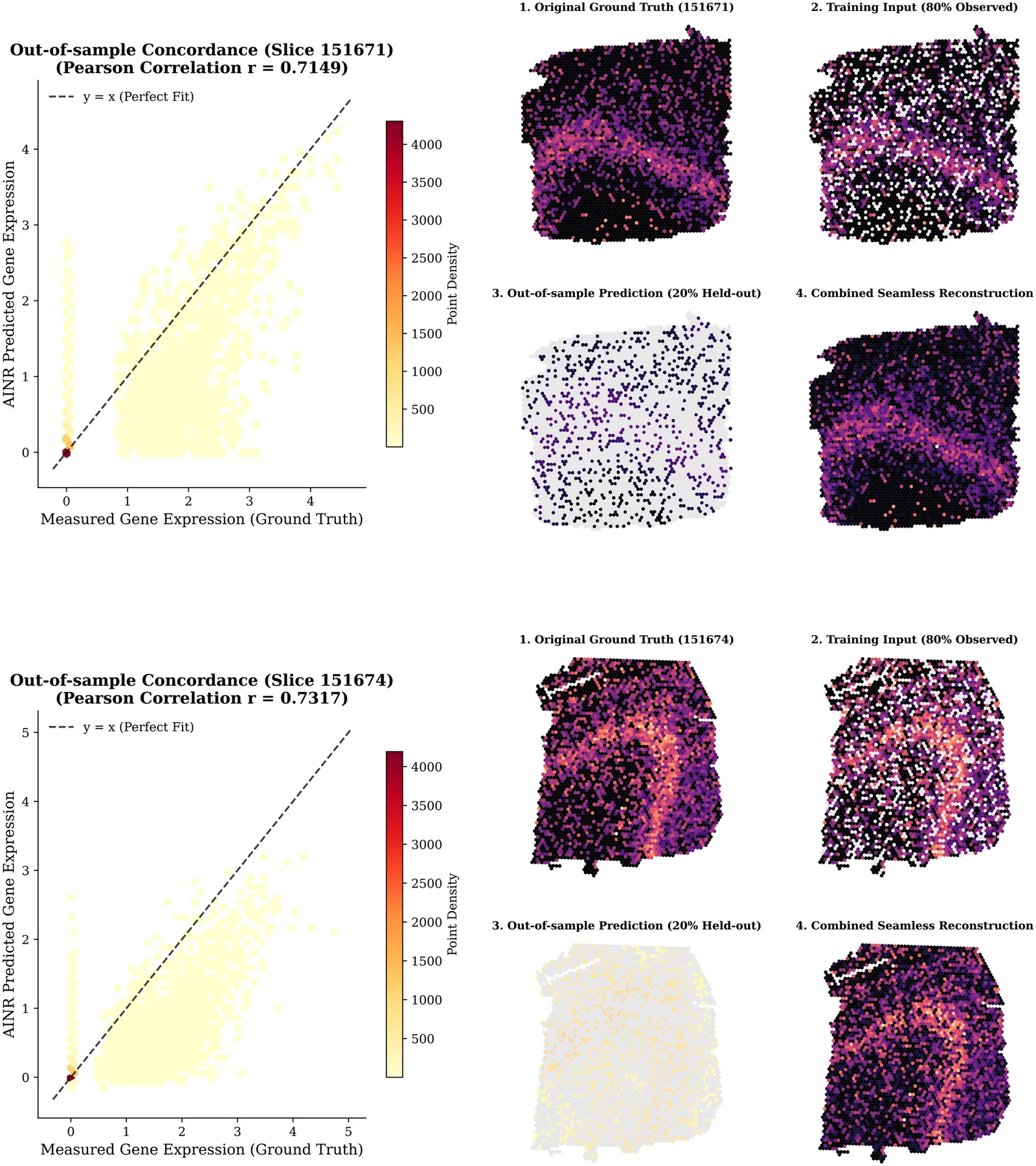
Validation of the continuous representation capability of AINR via out-of-sample prediction.

To evaluate whether AINR learns a genuine continuous biological field, we performed two complementary validation experiments: an out-of-sample concordance analysis and a spatial gradient magnitude (SGM) mapping. We implemented a held-out validation strategy by randomly partitioning the spatial spots of a tissue slice (e.g. Slice 151 671 and 151 674) into a training set (80%) and a test set (20%). The model was trained solely on the observed (80%) of spots, and the expression values for the remaining 20% were predicted by querying the learned continuous function $f_{\theta }$ at the corresponding coordinates. As illustrated in Fig. 10, AINR achieved a high Pearson correlation between the predicted and measured gene expressions. The “Combined Seamless Reconstruction” maps demonstrate that AINR can successfully recover the missing spatial signals with high fidelity, producing a continuous expression landscape that is visually indistinguishable from the original ground truth. This ability to generalize to unseen coordinates provides definitive evidence that AINR has captured the underlying continuous manifold of the transcriptome, transcending the limitations of discrete data interpolation.

**Figure 11:**
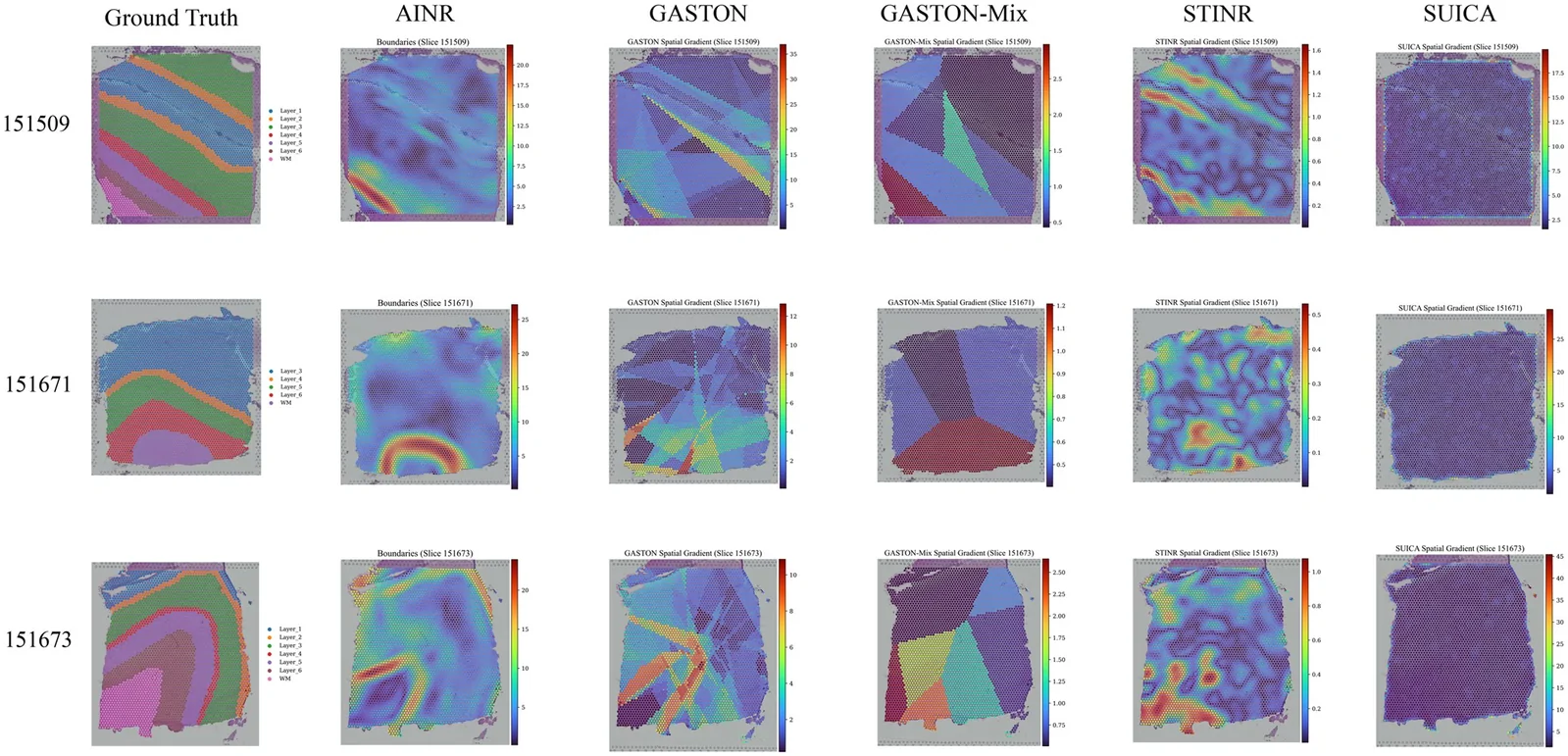
Identification of biological boundaries using Spatial Gradient Magnitude (SGM). For representative slices (151 509, 151 671, and 151 673).

Leveraging the differentiability of the INR framework, we further explored the capacity of AINR to autonomously identify anatomical transitions. We calculated the Spatial Gradient Magnitude (SGM) of the latent representations $Z_{\alpha }$ with respect to the spatial coordinates v, defined as $\text{SGM}(v)=∥\nabla_{v}Z_{\alpha }{∥}_{2}$. In theory, regions of high gradient intensity correspond to areas of rapid biological transition, which should align with the boundaries between distinct histological layers. As shown in Fig. 11, the SGM maps for multiple slices (e.g. 151 509, 151 671, and 151 673) exhibit high-intensity “ridges” that precisely coincide with the known anatomical boundaries of the cortical layers and the white matter (WM). In contrast to the cluster maps, which represent stable biological states, the SGM maps highlight the transition zones. The Spatial Gradient Magnitude (SGM) maps should be interpreted as maps of transition zones rather than direct maps of domain membership. For each slice, the high-intensity ridges localize where the transcriptomic field changes most rapidly, i.e. the boundaries between neighboring layers; within a layer’s interior the gradient is low, which is expected because the expression field is nearly constant there. We note, however, that the gradient contrast is not uniform across all boundaries. For example, in slice 151 671, the boundary around Layer 3 is comparatively weak and the corresponding SGM signal is less pronounced (Fig. 11). This is consistent with the biology of the DLPFC: the transcriptomes of Layers 2, 3 and 4 are closely related, so the transitions between them are gradual rather than abrupt. In contrast, sharp anatomical interfaces such as the white matter boundary, where the transcriptomic shift is abrupt, produce strong, well-defined SGM ridges. The SGM maps thus capture the degree of biological transition, and where a boundary is gradual (as around Layer 3 in slice 151 671), the corresponding gradient is proportionally weaker. This interpretation reconciles the visual appearance of the Layer 3 region with the known laminar anatomy of the cortex. This high spatial congruence between the calculated gradients and the ground truth boundaries demonstrates that the Lipschitz-constrained continuous field learned by AINR effectively captures the intrinsic geometric structure of the tissue. By transforming the domain identification problem into a gradient analysis task, AINR provides a robust and interpretable mechanism for delineating fine-grained biological boundaries without relying on explicit adjacency graphs. A systematic benchmark comparing AINR with other neural field methods (GASTON, SUICA, STINR, and GASTON-Mix) across all 12 DLPFC slices further confirmed that AINR achieved the highest spatial structure (Moran’s I = 0.970), while GASTON demonstrated the strongest boundary detection (Contrast Ratio = 1.087) (Fig. 11, detailed in Supplementary data).

## 4 Conclusion and discussion

Deciphering the spatial architecture of tissues requires precise domain identification. While traditional graph-based methods are often constrained by the computational bottlenecks of adjacency matrix construction and limited flexibility in sparse-data scenario, we presented AINR, an end-to-end, global context-aware framework that introduces a continuous spatial representation framework. By replacing discrete spatial graphs with an Implicit Neural Representation network, AINR establishes a non-linear mapping from coordinates to a continuous latent space, bypassing the need for explicit graph topologies. The integration of a periodic sine activation function effectively mitigates “spectral bias,” capturing the high-frequency spatial gradients that represent fine-grained biological boundaries. Coupled with Spatially-Aware Attention (SAA) the framework seamlessly integrates local spatial smoothness—enforced by Total Variation (TV) regularization—with global contextual interactions, yielding highly expressive and biologically meaningful representations. Our extensive validation across multiple datasets demonstrates that AINR significantly enhances the accuracy of spatial domain identification and effectively recovers the underlying biological continuum. It should be noted that the spatial smoothness prior inherent in the INR framework, while beneficial for suppressing noise in tissues with laminar or gradient-based architectures, may risk over-smoothing sharp transcriptomic boundaries in highly heterogeneous tissues such as aggressive tumor microenvironments; careful tuning of the TV regularization weight is recommended for such scenarios. However, several avenues for future research remain. While AINR circumvents the memory intensity of explicit graphs, the global attention mechanism may face scalability challenges as single-cell spatial datasets reach the million-spot scale. Future work will explore linear-complexity attention mechanisms and adaptive spatial down-sampling strategies to enhance scalability. Additionally, as spatial multi-omics matures, we intend to extend AINR to incorporate paired histological images and single-cell RNA sequencing (scRNA-seq) data, thereby refining boundary resolution and facilitating high-precision cell-type deconvolution. In this study, the number of clusters was primarily determined based on established biological priors. However, for tissues with unknown architectures, the optimal number of clusters in AINR can be estimated using standard unsupervised strategies, such as the Elbow method, by monitoring the sum of squared errors across a range of k values—or the Silhouette Coefficient, which measures the balance between cluster cohesion and separation. These heuristic methods enable AINR to be applied flexibly to a wide variety of novel tissue types. In conclusion, AINR provides a versatile and powerful framework for advancing spatial transcriptomics research across a broad spectrum of biological contexts.

## Supplementary Material

vbag241_Supplementary_Data

## Data Availability

The data used in this paper are all publicly available. Detailed information can be found in Table S1. The code for the AINR algorithm is deposited at https://github.com/XGD1122/AINR
