# Supplementary material for: AINR: attention-guided implicit neural representations for spatial domain identification in spatial transcriptomics
Source: Bioinform Adv. 2026 Aug 19;6(1):vbag241. doi: 10.1093/bioadv/vbag241 (PMC13590037; doi:10.1093/bioadv/vbag241)
Supplement: Supplementary file 1 [file vbag241_supplementary_data.zip › Figure S4.pdf]

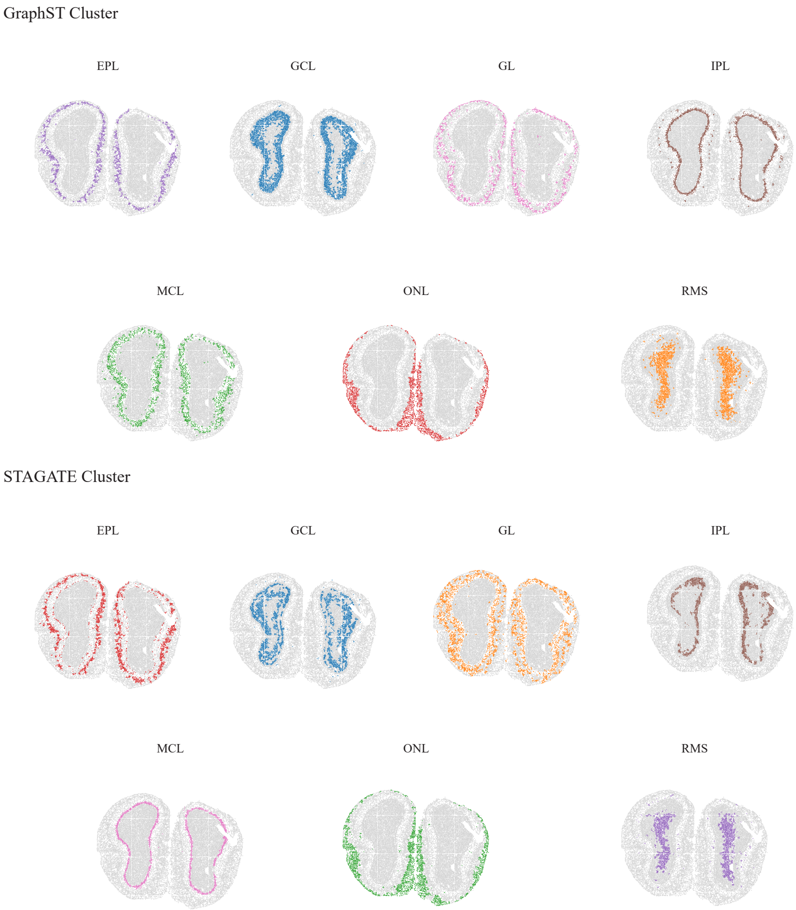
GraphST Cluster
EPL
GCL
GL
IPL
MCL
ONL
RMS
STAGATE Cluster
EPL
GCL
GL
IPL
MCL
ONL
RMS
