# Supplementary material for: AINR: attention-guided implicit neural representations for spatial domain identification in spatial transcriptomics
Source: Bioinform Adv. 2026 Aug 19;6(1):vbag241. doi: 10.1093/bioadv/vbag241 (PMC13590037; doi:10.1093/bioadv/vbag241)
Supplement: Supplementary file 1 [file vbag241_supplementary_data.zip › Figure S5.pdf]

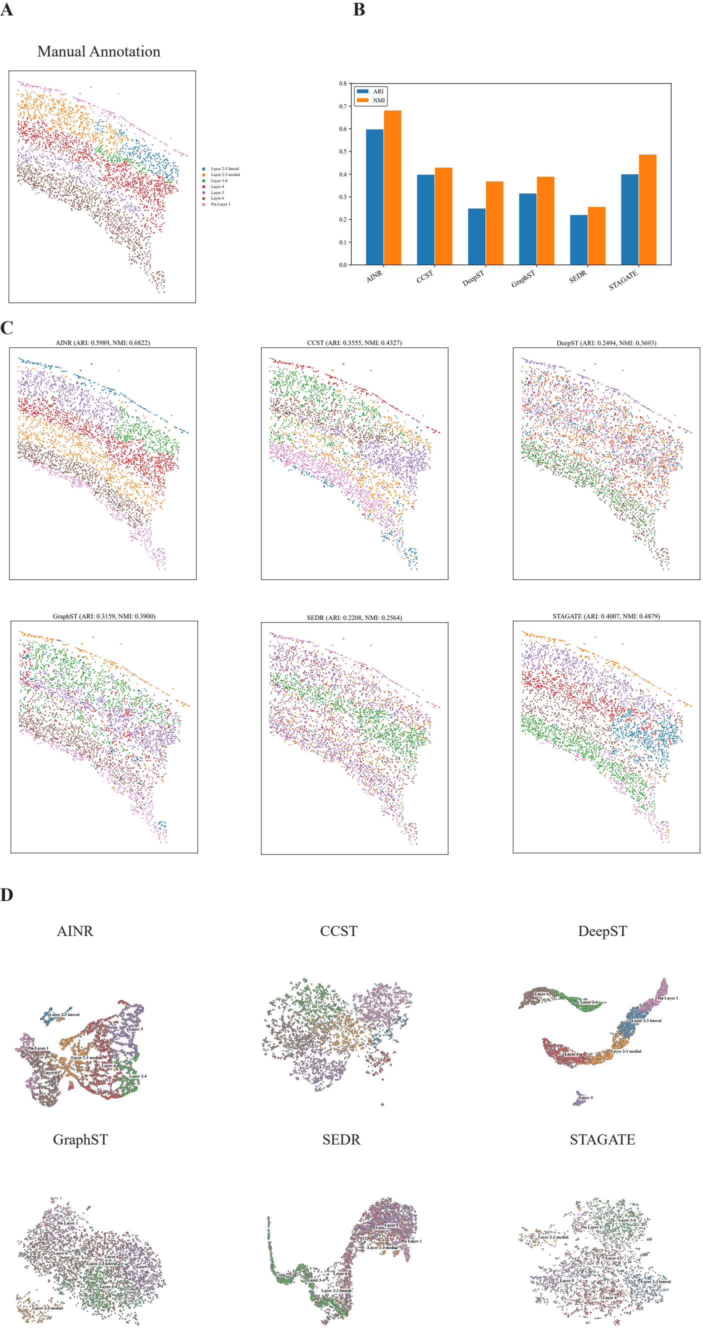
A
B
Manual Annotation
Layer 2-3 lateral
Layer 2-3 medial
Layer 3-4
Layer 4
Layer 5
Layer 6
Pia Layer 1
ARI
NMI
0.8
0.7
0.6
0.5
0.4
0.3
0.2
0.1
0.0
AINR
CCST
DeepST
GraphST
SEDR
STAGATE
C
AINR (ARI: 0.5989, NMI: 0.6822)
CCST (ARI: 0.3555, NMI: 0.4327)
DeepST (ARI: 0.2494, NMI: 0.3693)
GraphST (ARI: 0.3159, NMI: 0.3900)
SEDR (ARI: 0.2208, NMI: 0.2564)
STAGATE (ARI: 0.4007, NMI: 0.4879)
D
AINR
CCST
DeepST
GraphST
SEDR
STAGATE
