# Supplementary material for: AINR: attention-guided implicit neural representations for spatial domain identification in spatial transcriptomics
Source: Bioinform Adv. 2026 Aug 19;6(1):vbag241. doi: 10.1093/bioadv/vbag241 (PMC13590037; doi:10.1093/bioadv/vbag241)
Supplement: Supplementary file 1 [file vbag241_supplementary_data.zip › Figure S6.pdf]

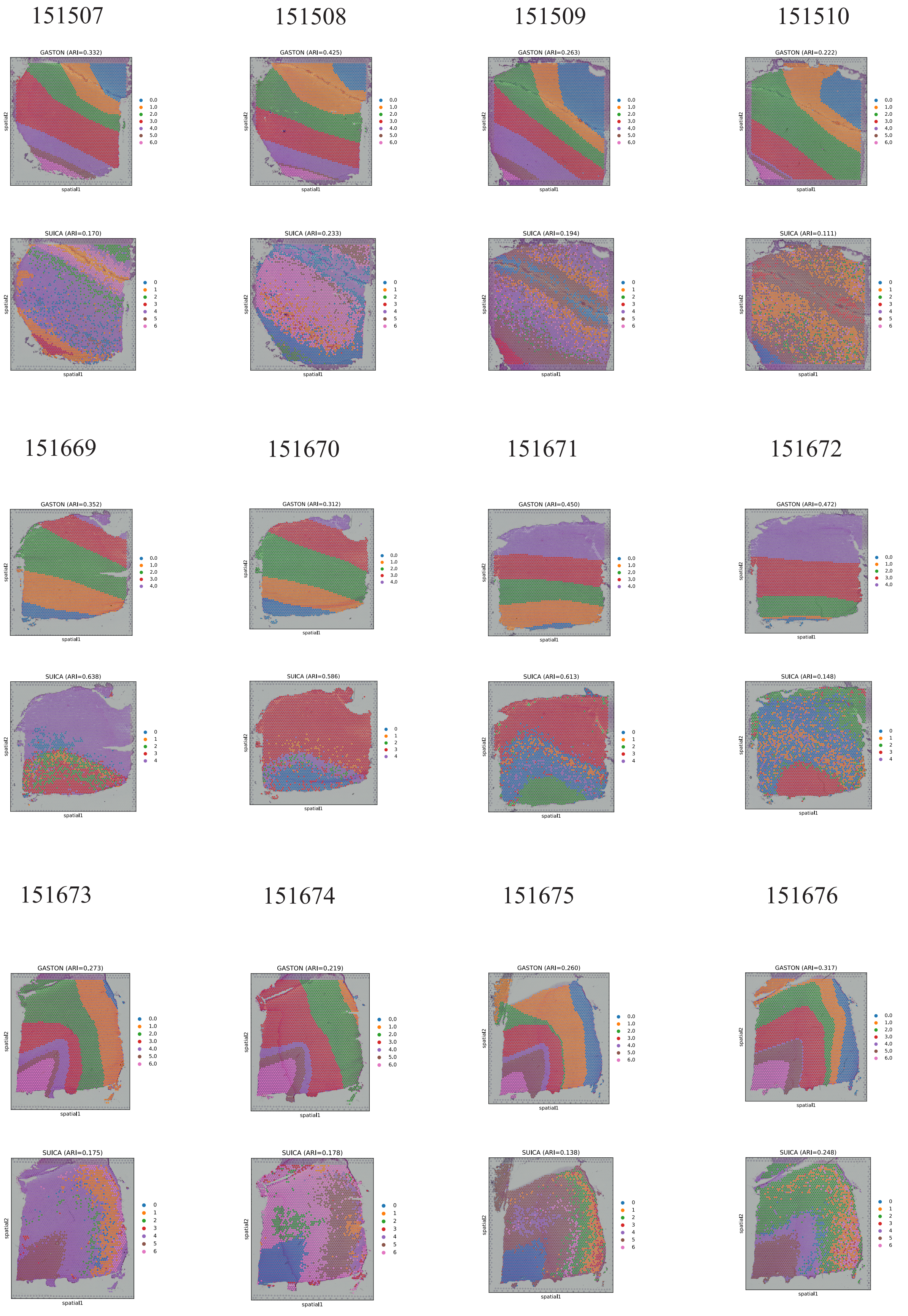
151507
GASTON (ARI=0.332)
SUICA (ARI=0.170)
151508
GASTON (ARI=0.425)
SUICA (ARI=0.233)
151509
GASTON (ARI=0.263)
SUICA (ARI=0.194)
151510
GASTON (ARI=0.222)
SUICA (ARI=0.111)
151669
GASTON (ARI=0.352)
SUICA (ARI=0.638)
151670
GASTON (ARI=0.312)
SUICA (ARI=0.586)
151671
GASTON (ARI=0.450)
SUICA (ARI=0.613)
151672
GASTON (ARI=0.472)
SUICA (ARI=0.148)
151673
GASTON (ARI=0.273)
SUICA (ARI=0.175)
151674
GASTON (ARI=0.219)
SUICA (ARI=0.178)
151675
GASTON (ARI=0.260)
SUICA (ARI=0.138)
151676
GASTON (ARI=0.317)
SUICA (ARI=0.248)
spatial1
spatial2
