# Supplementary material for: AINR: attention-guided implicit neural representations for spatial domain identification in spatial transcriptomics
Source: Bioinform Adv. 2026 Aug 19;6(1):vbag241. doi: 10.1093/bioadv/vbag241 (PMC13590037; doi:10.1093/bioadv/vbag241)
Supplement: Supplementary file 1 [file vbag241_supplementary_data.zip › Supplementary Material.docx]

# 1 Supplementary Information

## 1.1 S1. Data Details and Datasets Used for AINR

**Table S1.** Summary of spatial transcriptomics datasets used in this study.

| Platform | Tissue | Section | Spots | Genes | Source |
| --- | --- | --- | --- | --- | --- |
| 10x Visium | Human dorsolateral prefrontal cortex (DLPFC) | 151507 | 4226 | 33538 | http://spatial.libd.org/spatialLIBD/ |
| 10x Visium | Human dorsolateral prefrontal cortex (DLPFC) | 151508 | 4384 | 33538 | http://spatial.libd.org/spatialLIBD/ |
| 10x Visium | Human dorsolateral prefrontal cortex (DLPFC) | 151509 | 4789 | 33538 | http://spatial.libd.org/spatialLIBD/ |
| 10x Visium | Human dorsolateral prefrontal cortex (DLPFC) | 151510 | 4634 | 33538 | http://spatial.libd.org/spatialLIBD/ |
| 10x Visium | Human dorsolateral prefrontal cortex (DLPFC) | 151669 | 3661 | 33538 | http://spatial.libd.org/spatialLIBD/ |
| 10x Visium | Human dorsolateral prefrontal cortex (DLPFC) | 151670 | 3498 | 33538 | http://spatial.libd.org/spatialLIBD/ |
| 10x Visium | Human dorsolateral prefrontal cortex (DLPFC) | 151671 | 4110 | 33538 | http://spatial.libd.org/spatialLIBD/ |
| 10x Visium | Human dorsolateral prefrontal cortex (DLPFC) | 151672 | 4015 | 33538 | http://spatial.libd.org/spatialLIBD/ |
| 10x Visium | Human dorsolateral prefrontal cortex (DLPFC) | 151673 | 3639 | 33538 | http://spatial.libd.org/spatialLIBD/ |
| 10x Visium | Human dorsolateral prefrontal cortex (DLPFC) | 151674 | 3673 | 33538 | http://spatial.libd.org/spatialLIBD/ |
| 10x Visium | Human dorsolateral prefrontal cortex (DLPFC) | 151675 | 3592 | 33538 | http://spatial.libd.org/spatialLIBD/ |
| 10x Visium | Human dorsolateral prefrontal cortex (DLPFC) | 151676 | 3460 | 33538 | http://spatial.libd.org/spatialLIBD/ |
| 10x Visium | Human breast cancer | Section 1 V1.1.0 | 3798 | 36601 | https://www.10xgenomics.com/datasets/human-breast-cancer-block-a-section-1-1-standard-1-1-0 |
| Stereo-seq | Mouse olfactory bulb | Single | 19109 | 14376 | https://github.com/JinmiaoChenLab/SEDR_analyses |
| Slide-seqV2 | Mouse Hippocampus | Puck_200127_15 | 52869 | 23264 | https://singlecell.broadinstitute.org/single_cell/study/SCP354/slide-seq-study |
| STARmap | Mouse visual cortex | Single | 1207 | 1020 | https://drive.google.com/drive/folders/1I1nxheWlc2RXSdiv24dex3YRaEh780my |
| osmFISH | Mouse somatosensory cortex | Single | 3405 | 33 | https://linnarssonlab.org/osmFISH/ |

## 1.2 S2. Evaluation Metrics of AINR

The clustering performance is quantified using the Adjusted Rand Index (ARI) and Normalized Mutual Information (NMI). For boundary detection evaluation, we additionally employ three spatial gradient metrics: Moran’s I, Contrast Ratio, and AUROC.

### Adjusted Rand Index (ARI)

The ARI measures the similarity between the predicted clustering results and the ground-truth labels, adjusted for the chance grouping of elements:

$$ARI=\frac{\sum_{i,j} \binom{n_{ij}}{2}-\frac{[\sum_{i} \binom{a_{i}}{2}\sum_{j} \binom{b_{j}}{2}]}{\binom{n}{2}}}{\frac{1}{2}[\sum_{i} \binom{a_{i}}{2}+\sum_{j} \binom{b_{j}}{2}]-\frac{[\sum_{i} \binom{a_{i}}{2}\sum_{j} \binom{b_{j}}{2}]}{\binom{n}{2}}}$$

where $a_{i}$ and $b_{j}$ denote the number of samples in the $i$-th predicted cluster and the $j$-th true cluster, respectively.

### Normalized Mutual Information (NMI)

The NMI quantifies the mutual dependence between the predicted labels $\Omega$ and the ground-truth labels $C$:

$$NMI(\Omega,C)=\frac{2\times I(\Omega;C)}{H(\Omega)+H(C)}$$

where $I(\Omega;C)$ is the mutual information between the predicted clusters $\Omega$ and the ground-truth clusters $C$. The mutual information is calculated as follows:

$$I(\Omega;C)=\sum_{i=1}^{|\Omega|} \sum_{j=1}^{|C|} \frac{n_{ij}}{n}\log\frac{n\cdot n_{ij}}{a_{i}\cdot b_{j}}$$

### Spatial Gradient Evaluation Metrics

For evaluation of spatial gradient quality in the boundary detection benchmark (comparing neural field methods on their ability to identify anatomical transitions), we employ three complementary metrics:

**Moran’s I:** Measures the global spatial autocorrelation of the gradient magnitude field, indicating the degree of structured spatial variation. Higher values reflect stronger global spatial organization of the learned gradient field.

**Contrast Ratio (CR):** Defined as the ratio of mean gradient magnitude within ground-truth boundary regions to that in interior regions, quantifying the contrast between boundaries and homogeneous domains. Values $>1$ indicate boundary enhancement over baseline.

**Area Under ROC Curve (AUROC):** Evaluates how well the gradient magnitude map discriminates between annotated boundary and non-boundary spots, providing a threshold-independent assessment of boundary detection accuracy.

### ARI Comparison on DLPFC Dataset

**Table S2.** ARI performance comparison between GASTON, SUICA, and AINR on 12 DLPFC sections.

| Section | GASTON | SUICA | AINR (Ours) |
| --- | --- | --- | --- |
| 151507 | 0.332 | 0.170 | **0.5959** |
| 151508 | 0.423 | 0.233 | **0.6172** |
| 151509 | 0.265 | 0.194 | **0.7456** |
| 151510 | 0.222 | 0.111 | **0.7450** |
| 151669 | 0.252 | 0.438 | **0.7675** |
| 151670 | 0.313 | 0.585 | **0.7942** |
| 151671 | 0.450 | 0.613 | **0.8492** |
| 151672 | 0.472 | 0.140 | **0.8039** |
| 151673 | 0.272 | 0.175 | **0.6848** |
| 151674 | 0.239 | 0.170 | **0.7118** |
| 151675 | 0.260 | 0.138 | **0.6743** |
| 151676 | 0.317 | 0.248 | **0.6217** |

### Spatial Gradient Benchmark Comparison

To evaluate the ability of each neural field method to capture anatomical boundaries, we conducted a systematic gradient benchmark across all 12 DLPFC slices, comparing AINR with GASTON, SUICA, STINR, and GASTON-Mix. The benchmark evaluates three metrics—Moran’s I, Contrast Ratio, and AUROC—computed from the spatial gradient magnitude (SGM) of each model’s learned latent representation.

**Table S3.** Spatial gradient benchmark results across all 12 DLPFC slices (mean ± std). Bold indicates best performance. Higher Moran’s I, Contrast Ratio, and AUROC indicate stronger spatial structure and boundary detection.

| Model | Moran’s I | Contrast Ratio | AUROC |
| --- | --- | --- | --- |
| AINR (Ours) | **0.970** ± 0.012 | 1.045 ± 0.031 | 0.518 ± 0.042 |
| GASTON | 0.942 ± 0.018 | **1.087** ± 0.045 | **0.546** ± 0.038 |
| SUICA | 0.398 ± 0.089 | 0.921 ± 0.052 | 0.461 ± 0.028 |
| STINR | 0.861 ± 0.025 | 0.983 ± 0.038 | 0.489 ± 0.035 |
| GASTON-Mix | 0.523 ± 0.067 | 0.901 ± 0.041 | 0.452 ± 0.031 |

## 1.3 S3. Experimental Settings

All experiments were implemented using PyTorch and trained on a server with an NVIDIA GPU 3060. For all datasets, including the DLPFC sections and the BRCA tissue, we employed a learning rate of 0.001 and the Adam optimizer. The depth of the network was set to 2, and the width was set to 128 by default. For the DLPFC dataset, the total execution time for all 12 sections was 750.09 s, with a maximum GPU memory consumption of 4266.88 MB.

## 1.4 Supplementary Figures

**Figure S1.** Visualization of six clustering methods on DLPFC data section (151507–151510).

**Figure S2.** Visualization of six clustering methods on DLPFC data section (151669–151672).

**Figure S3.** Visualization of six clustering methods on DLPFC data section (151673–151676).

**Figure S4.** Visualization of individual clustering results of GraphST and STAGATE in the mouse olfactory bulb dataset.

**Figure S5.** Spatial domains identification on the MSC osmFISH dataset. (A) Manual annotation. (B) ARI/NMI bar charts. (C) Identified spatial domains. (D) UMAP and PAGA graph.

**Figure S6.** Other INR method clustering result on DLPFC datasets.
