# Supplementary material for: AINR: attention-guided implicit neural representations for spatial domain identification in spatial transcriptomics
Source: Bioinform Adv. 2026 Aug 19;6(1):vbag241. doi: 10.1093/bioadv/vbag241 (PMC13590037; doi:10.1093/bioadv/vbag241)
Supplement: Supplementary file 1 [file vbag241_supplementary_data.zip › Figure S1.pdf]

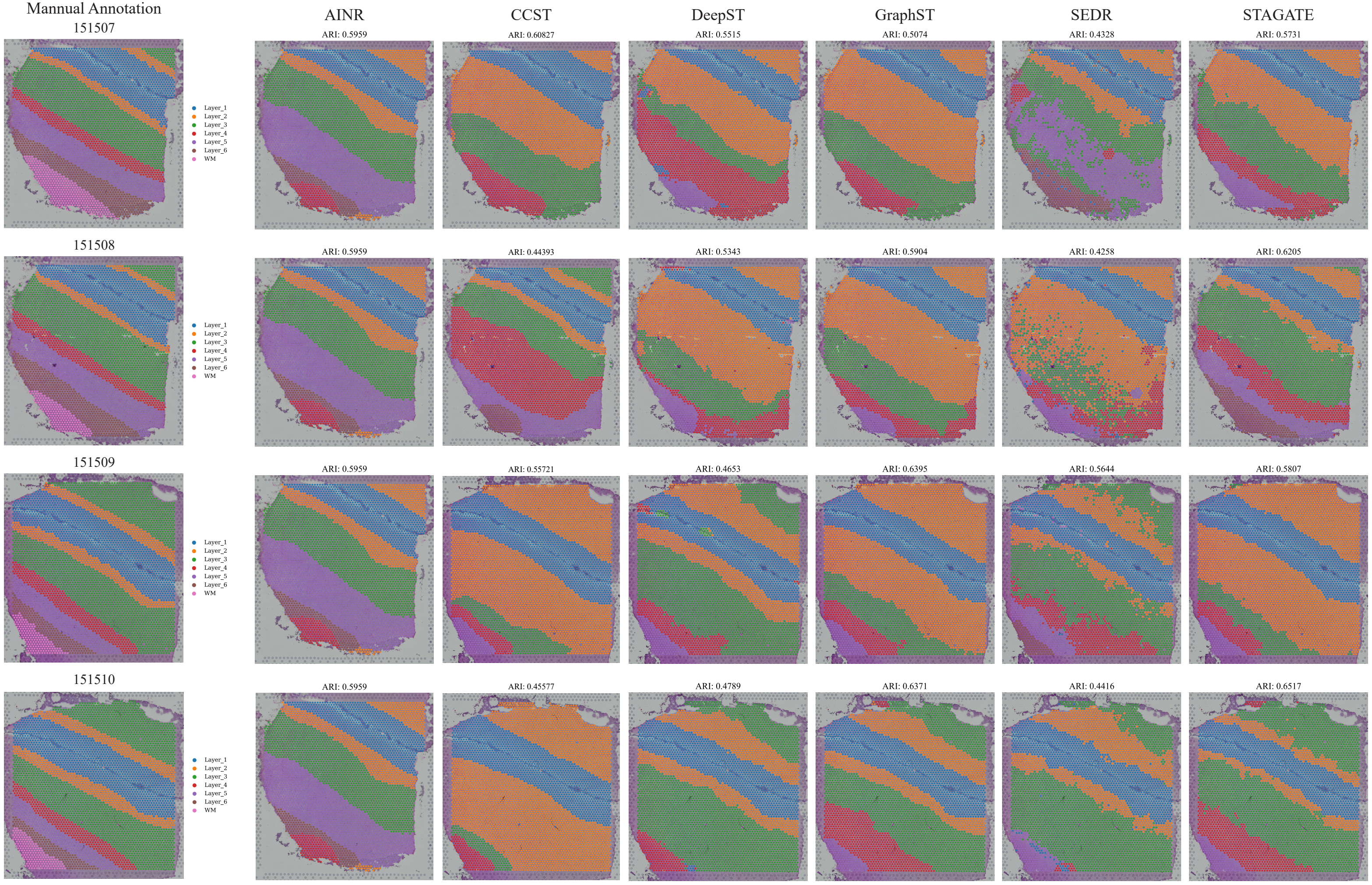
Mannual Annotation
AINR
CCST
DeepST
GraphST
SEDR
STAGATE
151507
Layer_1
Layer_2
Layer_3
Layer_4
Layer_5
Layer_6
WM
ARI: 0.5959
ARI: 0.60827
ARI: 0.5515
ARI: 0.5074
ARI: 0.4328
ARI: 0.5731
151508
Layer_1
Layer_2
Layer_3
Layer_4
Layer_5
Layer_6
WM
ARI: 0.5959
ARI: 0.44393
ARI: 0.5343
ARI: 0.5904
ARI: 0.4258
ARI: 0.6205
151509
Layer_1
Layer_2
Layer_3
Layer_4
Layer_5
Layer_6
WM
ARI: 0.5959
ARI: 0.55721
ARI: 0.4653
ARI: 0.6395
ARI: 0.5644
ARI: 0.5807
151510
Layer_1
Layer_2
Layer_3
Layer_4
Layer_5
Layer_6
WM
ARI: 0.5959
ARI: 0.45577
ARI: 0.4789
ARI: 0.6371
ARI: 0.4416
ARI: 0.6517
