# Supplementary material for: AINR: attention-guided implicit neural representations for spatial domain identification in spatial transcriptomics
Source: Bioinform Adv. 2026 Aug 19;6(1):vbag241. doi: 10.1093/bioadv/vbag241 (PMC13590037; doi:10.1093/bioadv/vbag241)
Supplement: Supplementary file 1 [file vbag241_supplementary_data.zip › Figure S2.pdf]

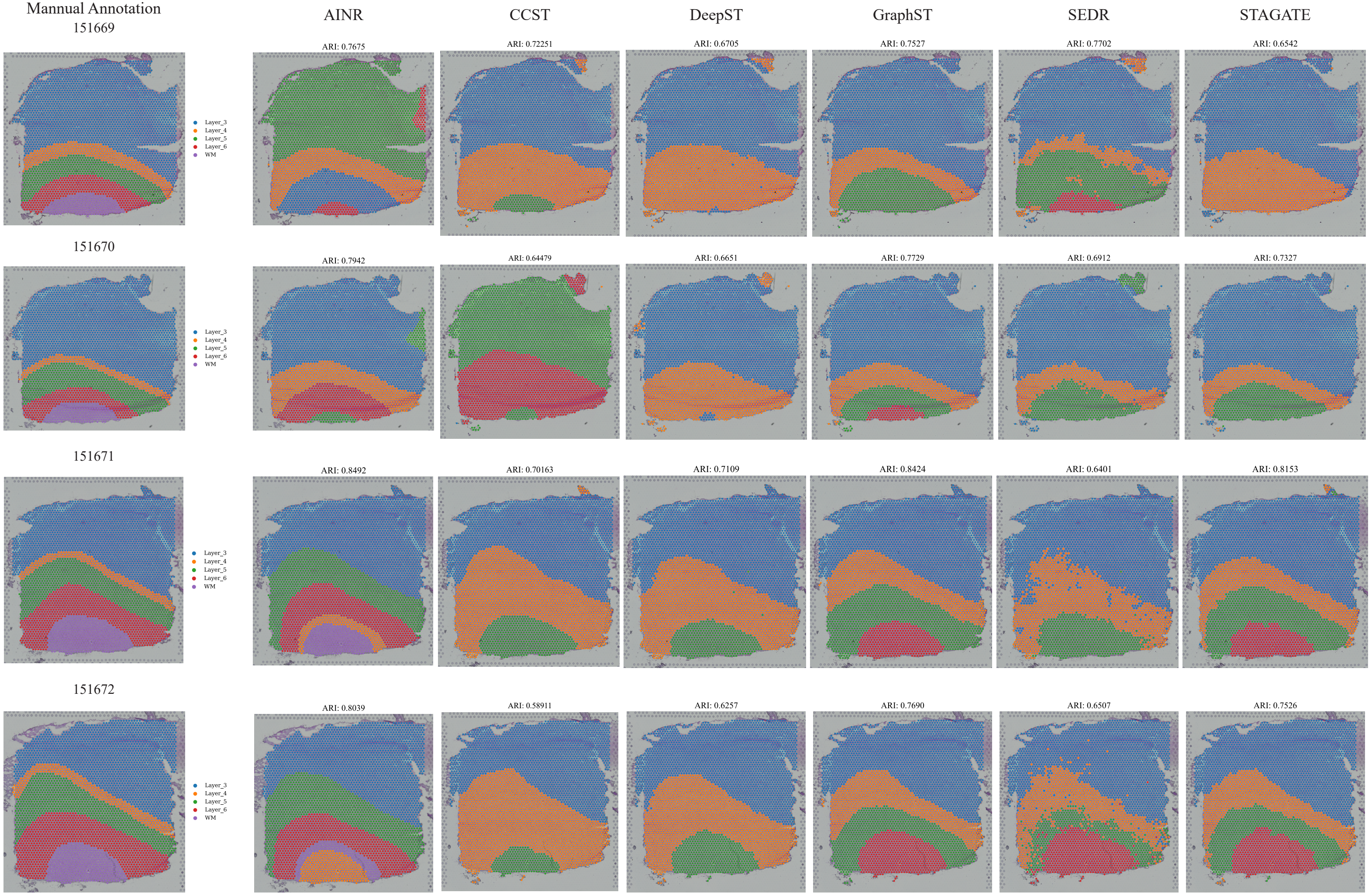

Mannual Annotation
AINR
CCST
DeepST
GraphST
SEDR
STAGATE
151669
ARI: 0.7675
ARI: 0.72251
ARI: 0.6705
ARI: 0.7527
ARI: 0.7702
ARI: 0.6542
151670
ARI: 0.7942
ARI: 0.64479
ARI: 0.6651
ARI: 0.7729
ARI: 0.6912
ARI: 0.7327
151671
ARI: 0.8492
ARI: 0.70163
ARI: 0.7109
ARI: 0.8424
ARI: 0.6401
ARI: 0.8153
151672
ARI: 0.8039
ARI: 0.58911
ARI: 0.6257
ARI: 0.7690
ARI: 0.6507
ARI: 0.7526
Layer_3
Layer_4
Layer_5
Layer_6
WM
