# Supplementary material for: AINR: attention-guided implicit neural representations for spatial domain identification in spatial transcriptomics
Source: Bioinform Adv. 2026 Aug 19;6(1):vbag241. doi: 10.1093/bioadv/vbag241 (PMC13590037; doi:10.1093/bioadv/vbag241)
Supplement: Supplementary file 1 [file vbag241_supplementary_data.zip › Figure S3.pdf]

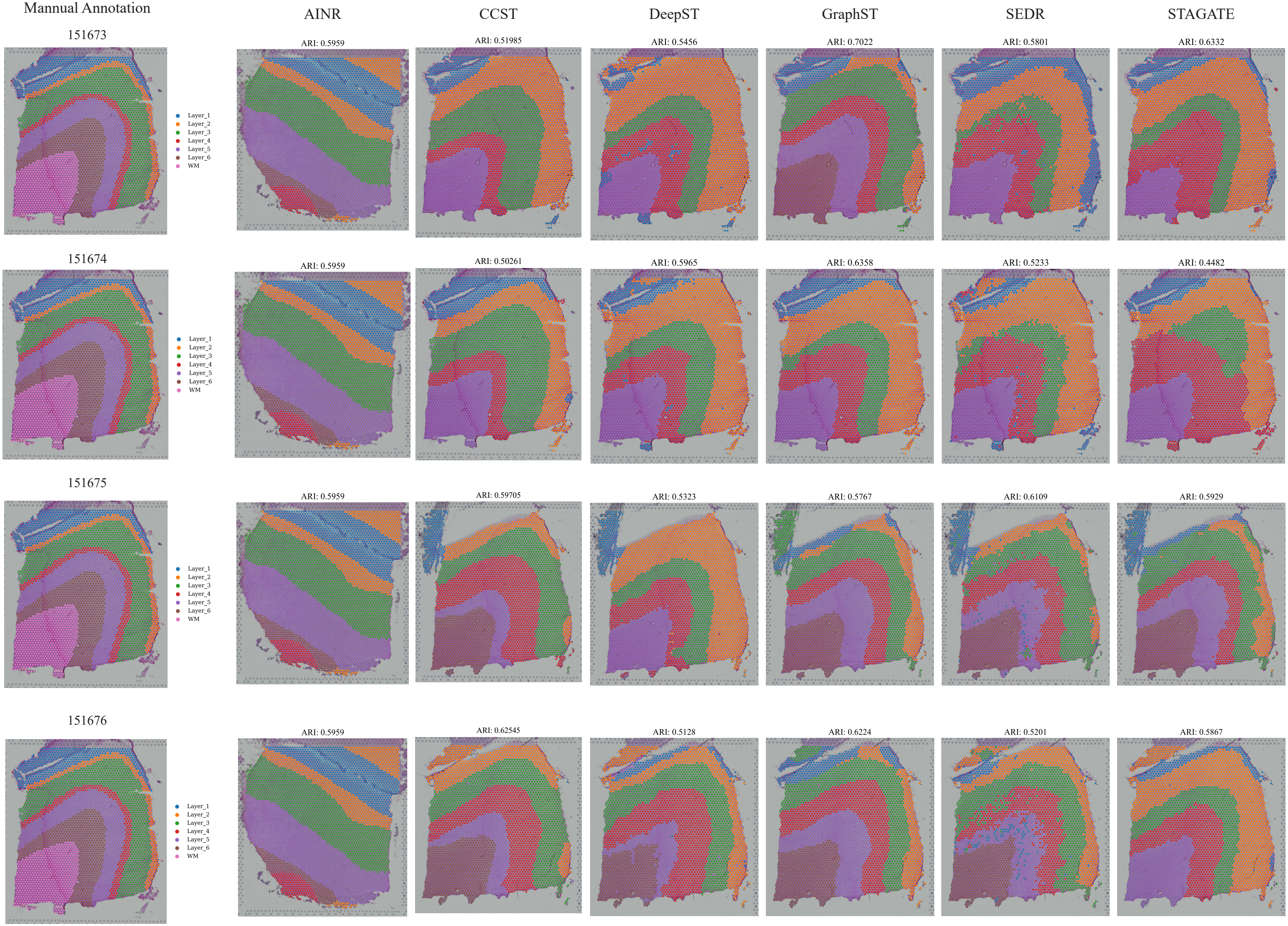

Mannual Annotation
AINR
CCST
DeepST
GraphST
SEDR
STAGATE
151673
ARI: 0.5959
ARI: 0.51985
ARI: 0.5456
ARI: 0.7022
ARI: 0.5801
ARI: 0.6332
151674
ARI: 0.5959
ARI: 0.50261
ARI: 0.5965
ARI: 0.6358
ARI: 0.5233
ARI: 0.4482
151675
ARI: 0.5959
ARI: 0.59705
ARI: 0.5323
ARI: 0.5767
ARI: 0.6109
ARI: 0.5929
151676
ARI: 0.5959
ARI: 0.62545
ARI: 0.5128
ARI: 0.6224
ARI: 0.5201
ARI: 0.5867
Layer_1
Layer_2
Layer_3
Layer_4
Layer_5
Layer_6
WM
